# Supplementary figures and images for: Recombinant fusion protein by lysozyme and antibacterial peptide enhances ischemic wound healing via angiogenesis and reduction of inflammation in diabetic db/db mice
Source: PeerJ. 2021 Apr 16;9:e11256. doi: 10.7717/peerj.11256 (PMC8054754; doi:10.7717/peerj.11256)

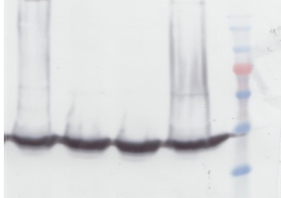

Supplement: Supplemental Information 2 — Representative images of granulation tissue sections. (A) Staining using Masson’s trichrome of ischemic and non-ischemic wounds in diabetic mice (×400). (B) HE staining of ischemic and non-ischemic wounds in diabetic mice (×400). (C) HE staining of ischemic and non-ischemic wounds in diabetic mice (×1,000). (D) Caspase-3 staining of ischemic and non-ischemic wounds in diabetic mice (×400). Scale bar = 100μm. (E) Duration of wound healing. [file peerj-09-11256-s002.zip › raw data 1/actin 1.png]

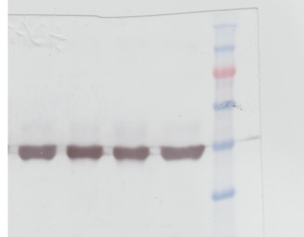

Supplement: Supplemental Information 2 — Representative images of granulation tissue sections. (A) Staining using Masson’s trichrome of ischemic and non-ischemic wounds in diabetic mice (×400). (B) HE staining of ischemic and non-ischemic wounds in diabetic mice (×400). (C) HE staining of ischemic and non-ischemic wounds in diabetic mice (×1,000). (D) Caspase-3 staining of ischemic and non-ischemic wounds in diabetic mice (×400). Scale bar = 100μm. (E) Duration of wound healing. [file peerj-09-11256-s002.zip › raw data 1/actin.png]

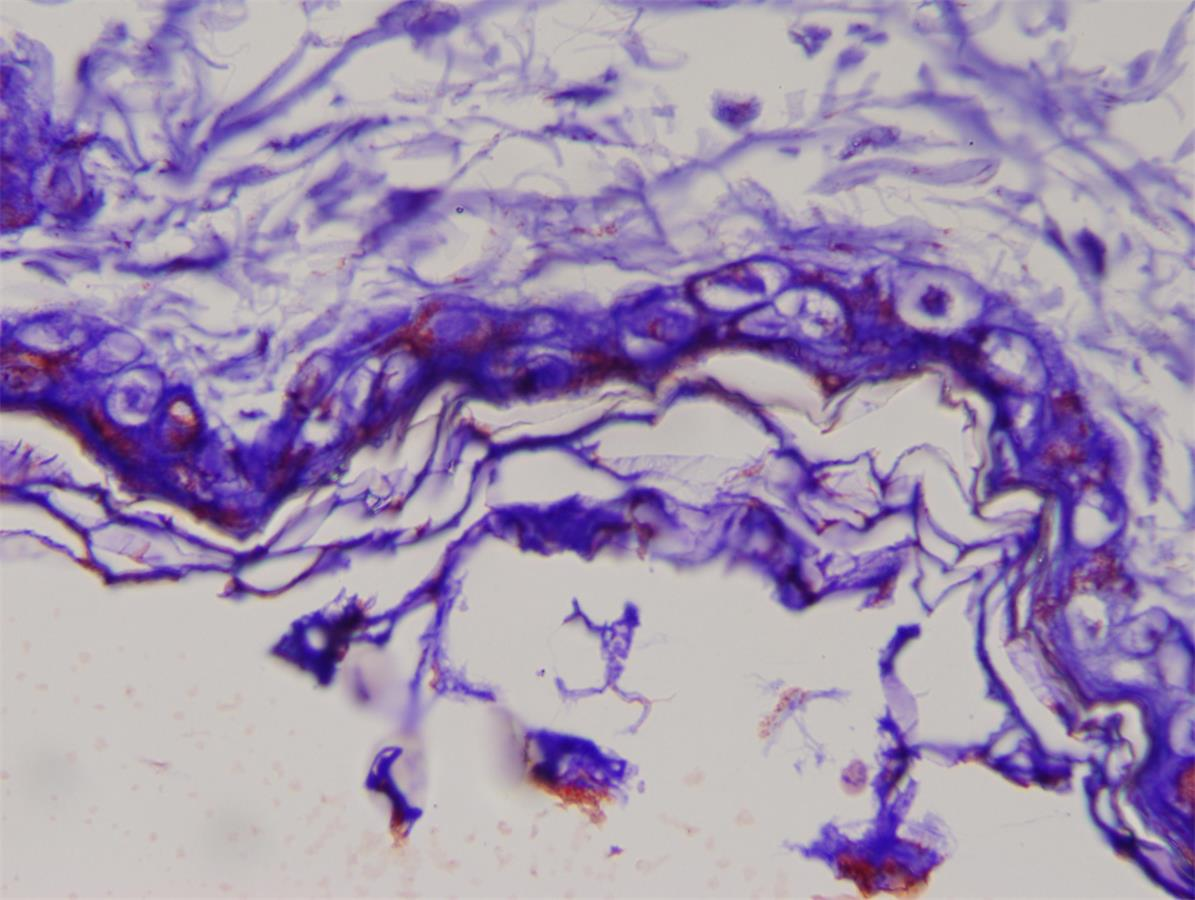

Supplement: Supplemental Information 2 — Representative images of granulation tissue sections. (A) Staining using Masson’s trichrome of ischemic and non-ischemic wounds in diabetic mice (×400). (B) HE staining of ischemic and non-ischemic wounds in diabetic mice (×400). (C) HE staining of ischemic and non-ischemic wounds in diabetic mice (×1,000). (D) Caspase-3 staining of ischemic and non-ischemic wounds in diabetic mice (×400). Scale bar = 100μm. (E) Duration of wound healing. [file peerj-09-11256-s002.zip › raw data 1/Figure 1A ISCH-vehicle.png]

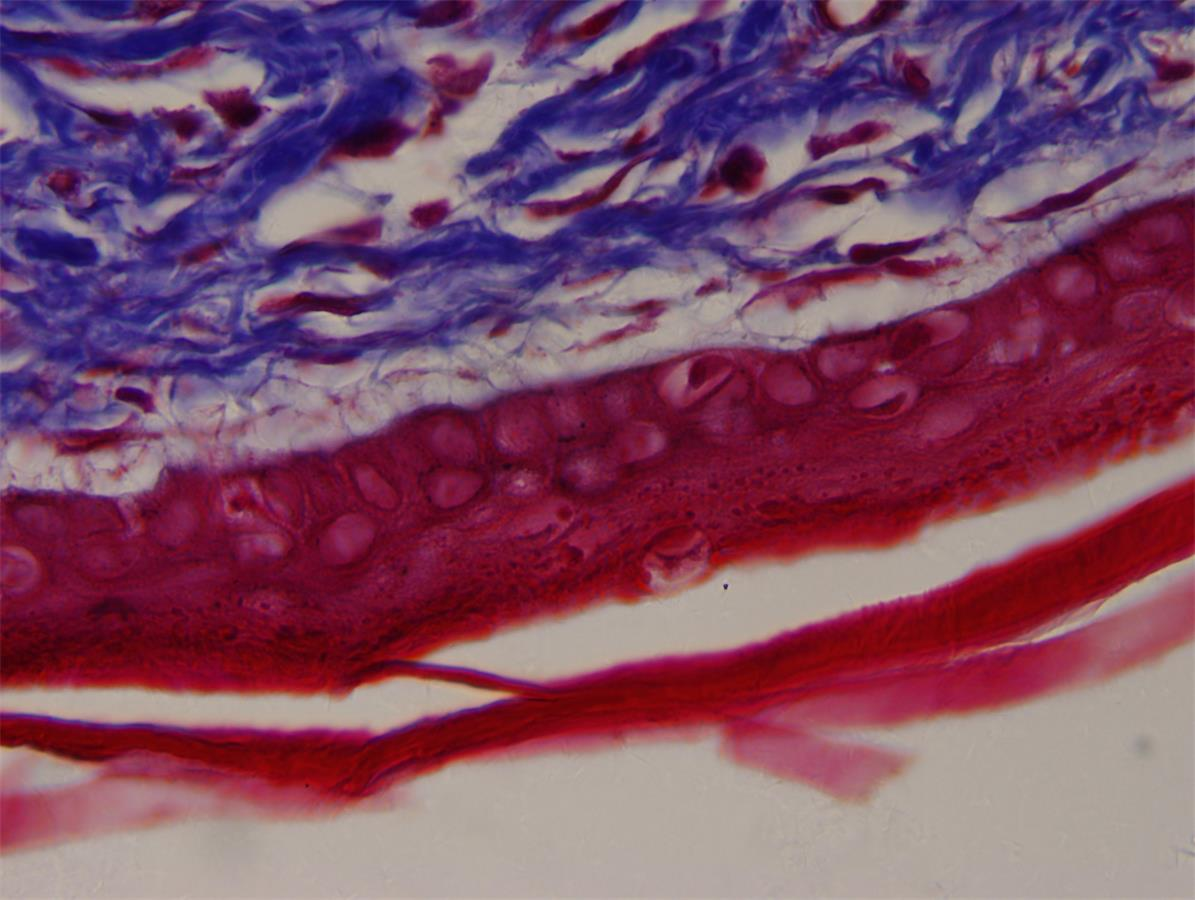

Supplement: Supplemental Information 2 — Representative images of granulation tissue sections. (A) Staining using Masson’s trichrome of ischemic and non-ischemic wounds in diabetic mice (×400). (B) HE staining of ischemic and non-ischemic wounds in diabetic mice (×400). (C) HE staining of ischemic and non-ischemic wounds in diabetic mice (×1,000). (D) Caspase-3 staining of ischemic and non-ischemic wounds in diabetic mice (×400). Scale bar = 100μm. (E) Duration of wound healing. [file peerj-09-11256-s002.zip › raw data 1/Figure 1A NONISCH-vehicle.png]

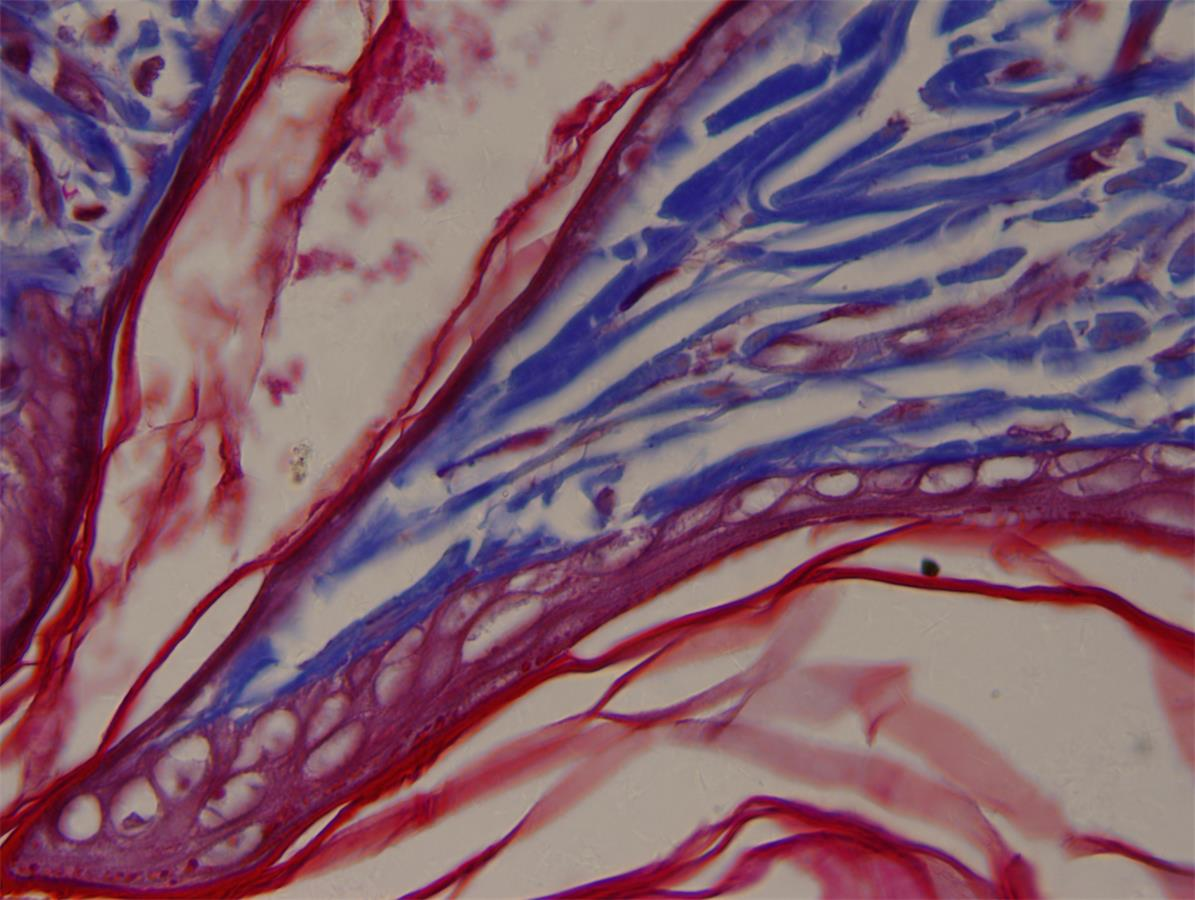

Supplement: Supplemental Information 2 — Representative images of granulation tissue sections. (A) Staining using Masson’s trichrome of ischemic and non-ischemic wounds in diabetic mice (×400). (B) HE staining of ischemic and non-ischemic wounds in diabetic mice (×400). (C) HE staining of ischemic and non-ischemic wounds in diabetic mice (×1,000). (D) Caspase-3 staining of ischemic and non-ischemic wounds in diabetic mice (×400). Scale bar = 100μm. (E) Duration of wound healing. [file peerj-09-11256-s002.zip › raw data 1/Figure 1A ISCH-fusion protein.png]

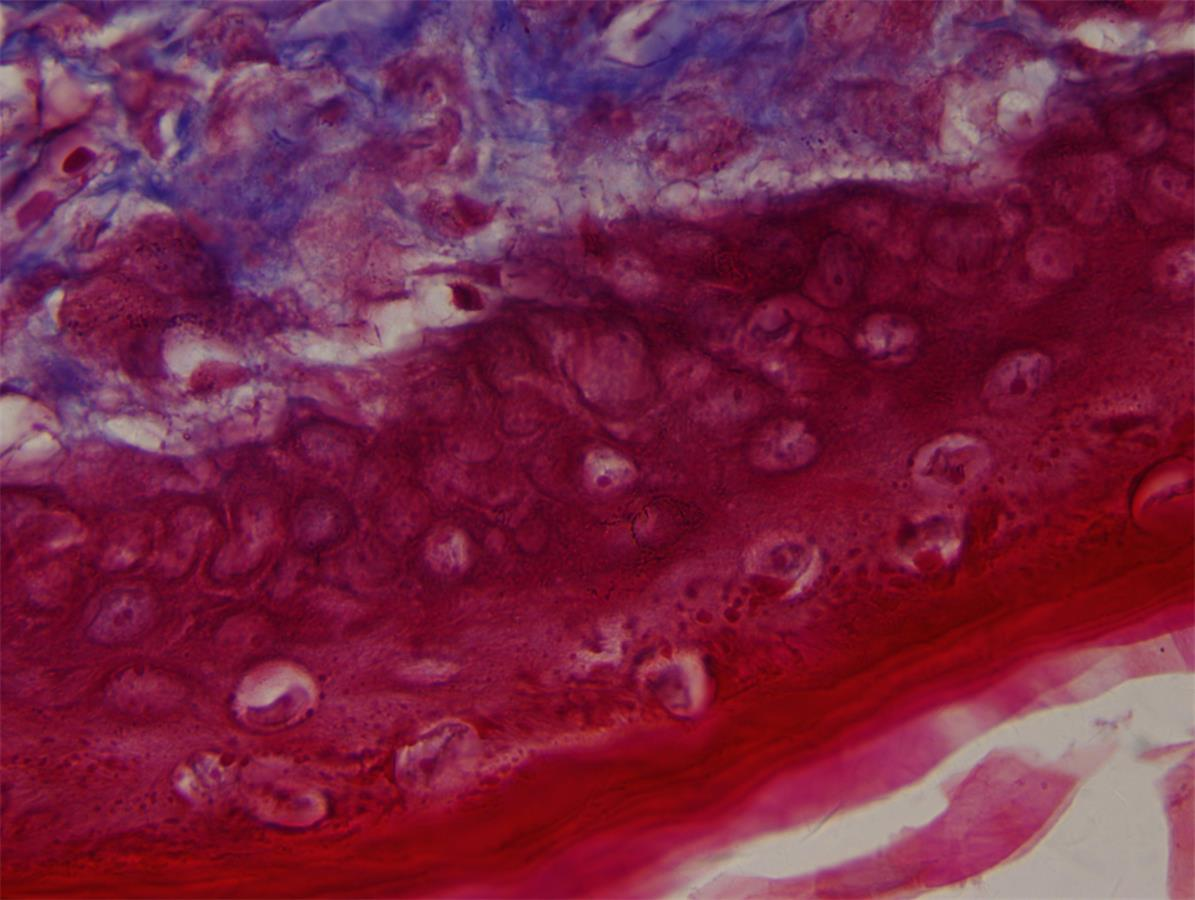

Supplement: Supplemental Information 2 — Representative images of granulation tissue sections. (A) Staining using Masson’s trichrome of ischemic and non-ischemic wounds in diabetic mice (×400). (B) HE staining of ischemic and non-ischemic wounds in diabetic mice (×400). (C) HE staining of ischemic and non-ischemic wounds in diabetic mice (×1,000). (D) Caspase-3 staining of ischemic and non-ischemic wounds in diabetic mice (×400). Scale bar = 100μm. (E) Duration of wound healing. [file peerj-09-11256-s002.zip › raw data 1/Figure 1A NONISCH-fusion protein.png]

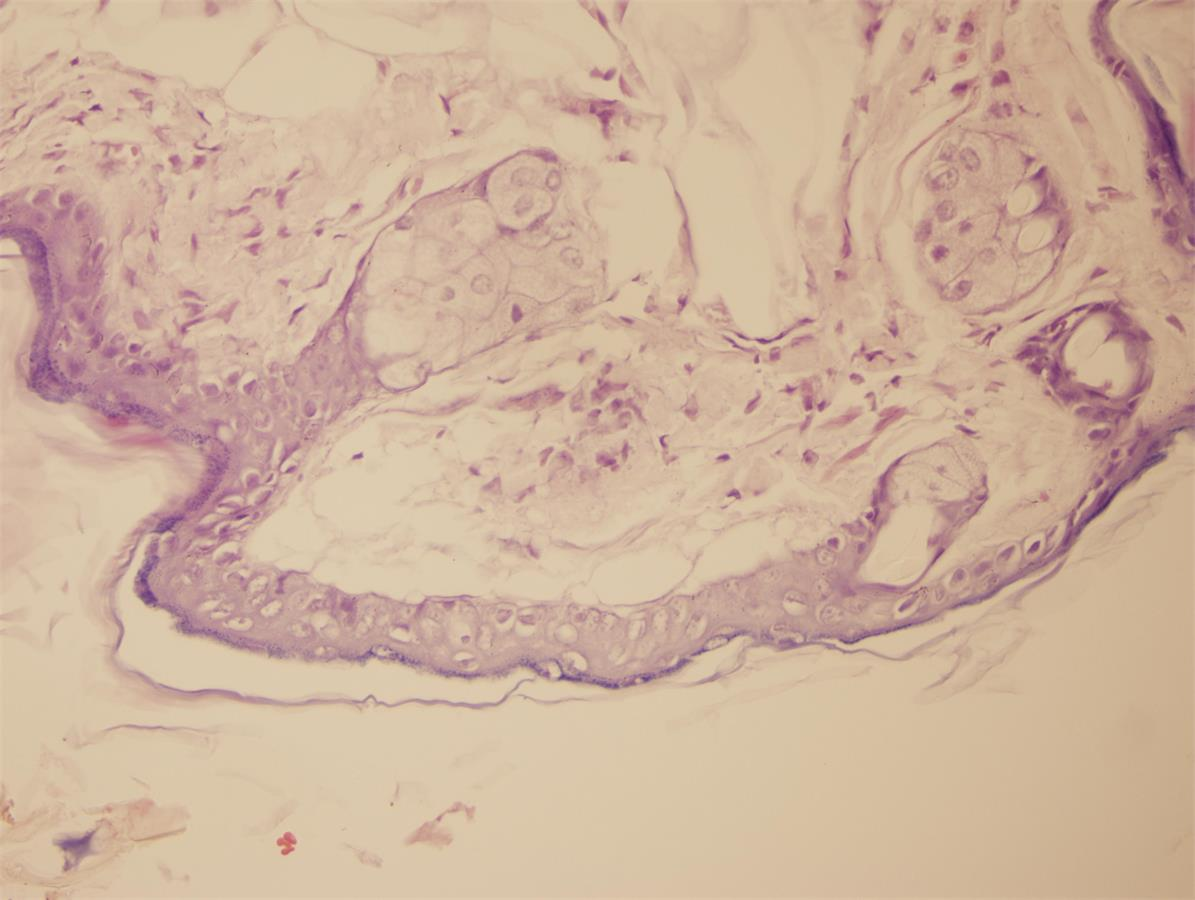

Supplement: Supplemental Information 2 — Representative images of granulation tissue sections. (A) Staining using Masson’s trichrome of ischemic and non-ischemic wounds in diabetic mice (×400). (B) HE staining of ischemic and non-ischemic wounds in diabetic mice (×400). (C) HE staining of ischemic and non-ischemic wounds in diabetic mice (×1,000). (D) Caspase-3 staining of ischemic and non-ischemic wounds in diabetic mice (×400). Scale bar = 100μm. (E) Duration of wound healing. [file peerj-09-11256-s002.zip › raw data 1/Figure 1B ISCH-vehicle.png]

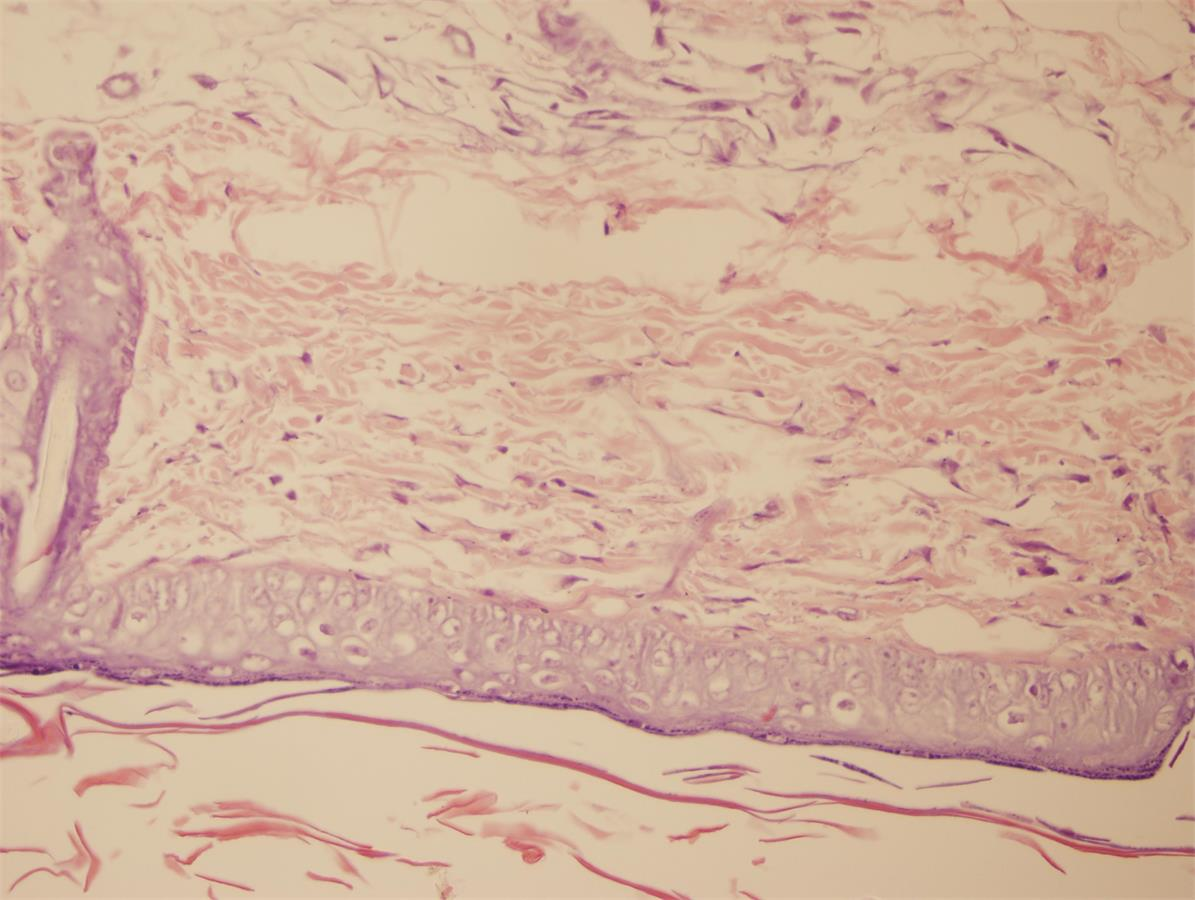

Supplement: Supplemental Information 2 — Representative images of granulation tissue sections. (A) Staining using Masson’s trichrome of ischemic and non-ischemic wounds in diabetic mice (×400). (B) HE staining of ischemic and non-ischemic wounds in diabetic mice (×400). (C) HE staining of ischemic and non-ischemic wounds in diabetic mice (×1,000). (D) Caspase-3 staining of ischemic and non-ischemic wounds in diabetic mice (×400). Scale bar = 100μm. (E) Duration of wound healing. [file peerj-09-11256-s002.zip › raw data 1/Figure 1B NONISCH-vehicle.png]

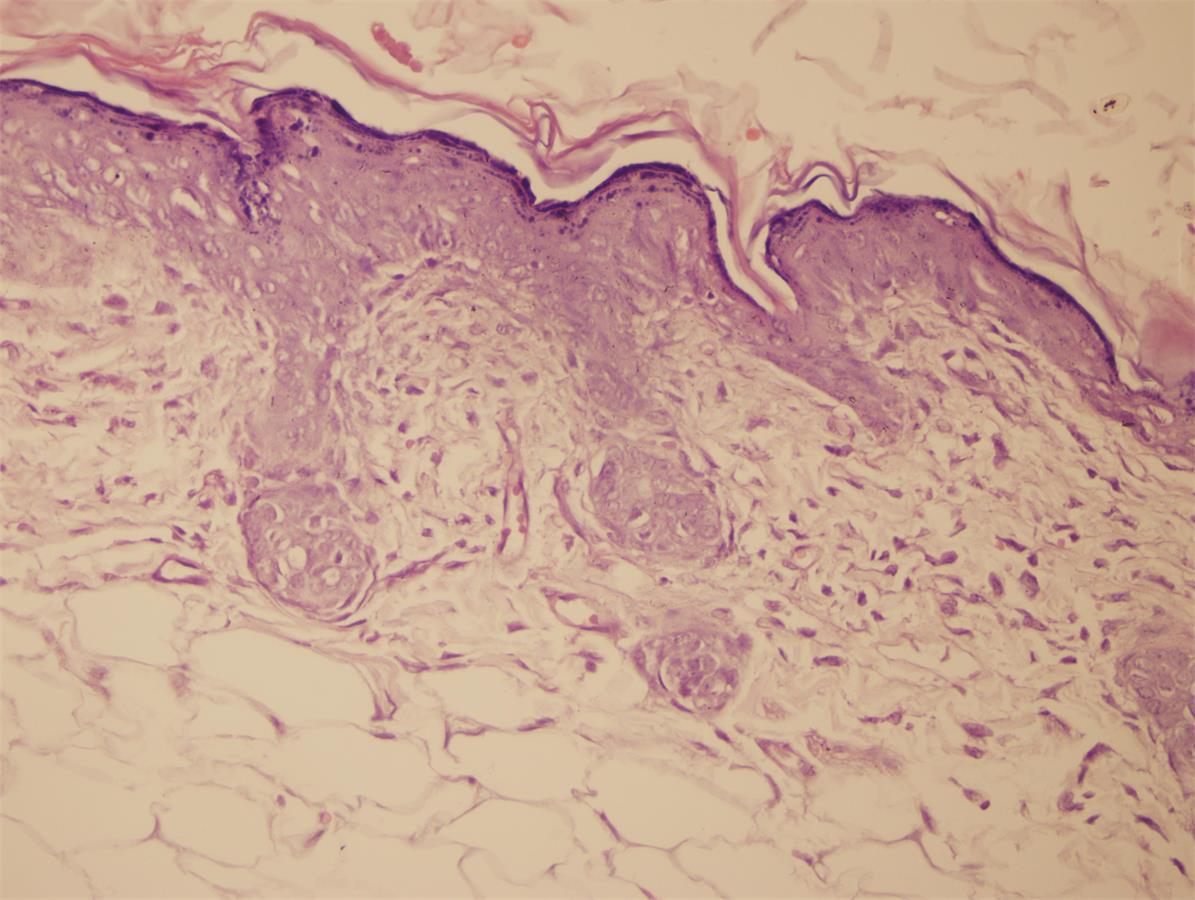

Supplement: Supplemental Information 2 — Representative images of granulation tissue sections. (A) Staining using Masson’s trichrome of ischemic and non-ischemic wounds in diabetic mice (×400). (B) HE staining of ischemic and non-ischemic wounds in diabetic mice (×400). (C) HE staining of ischemic and non-ischemic wounds in diabetic mice (×1,000). (D) Caspase-3 staining of ischemic and non-ischemic wounds in diabetic mice (×400). Scale bar = 100μm. (E) Duration of wound healing. [file peerj-09-11256-s002.zip › raw data 1/Figure 1B ISCH-fusion protein.png]

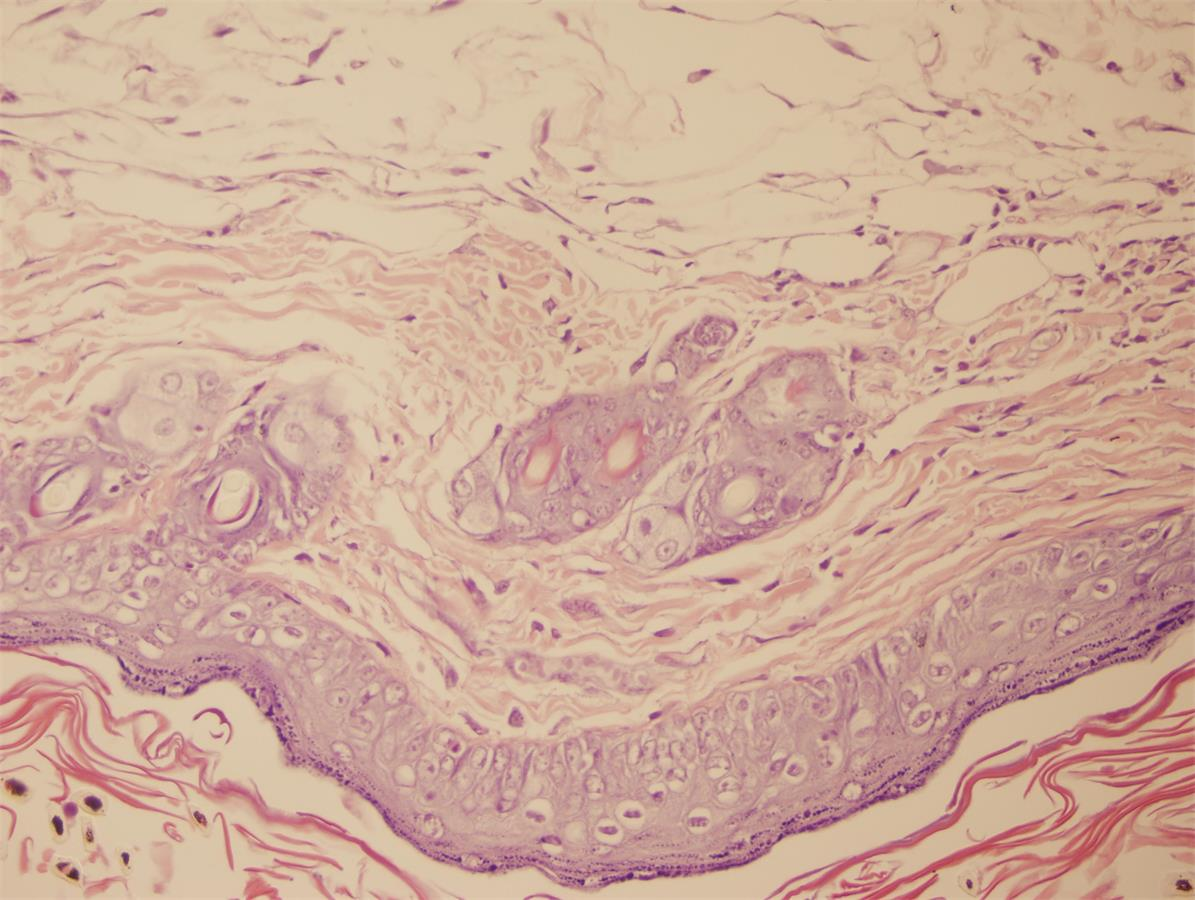

Supplement: Supplemental Information 2 — Representative images of granulation tissue sections. (A) Staining using Masson’s trichrome of ischemic and non-ischemic wounds in diabetic mice (×400). (B) HE staining of ischemic and non-ischemic wounds in diabetic mice (×400). (C) HE staining of ischemic and non-ischemic wounds in diabetic mice (×1,000). (D) Caspase-3 staining of ischemic and non-ischemic wounds in diabetic mice (×400). Scale bar = 100μm. (E) Duration of wound healing. [file peerj-09-11256-s002.zip › raw data 1/Figure 1B NONISCH-fusion protein.png]

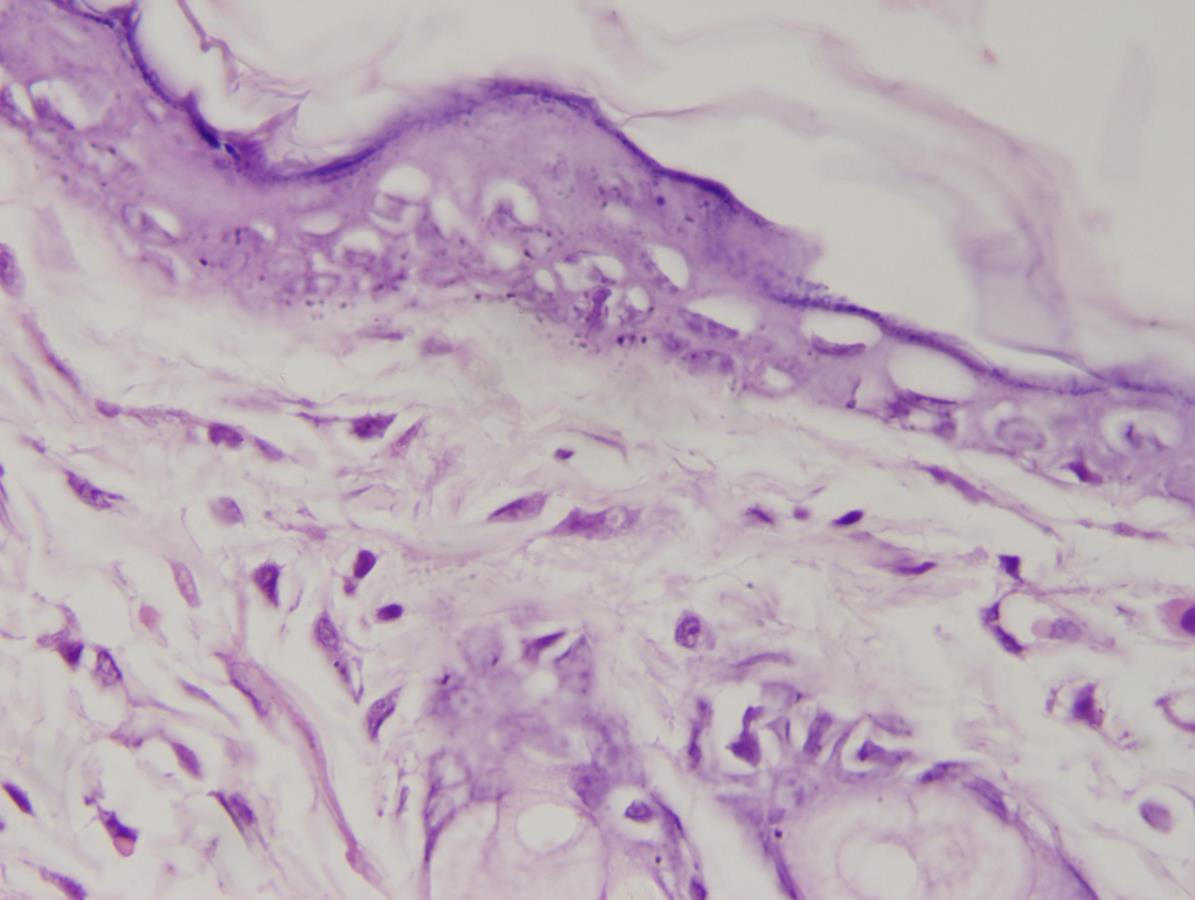

Supplement: Supplemental Information 2 — Representative images of granulation tissue sections. (A) Staining using Masson’s trichrome of ischemic and non-ischemic wounds in diabetic mice (×400). (B) HE staining of ischemic and non-ischemic wounds in diabetic mice (×400). (C) HE staining of ischemic and non-ischemic wounds in diabetic mice (×1,000). (D) Caspase-3 staining of ischemic and non-ischemic wounds in diabetic mice (×400). Scale bar = 100μm. (E) Duration of wound healing. [file peerj-09-11256-s002.zip › raw data 1/Figure 1C ISCH-vehicle.png]

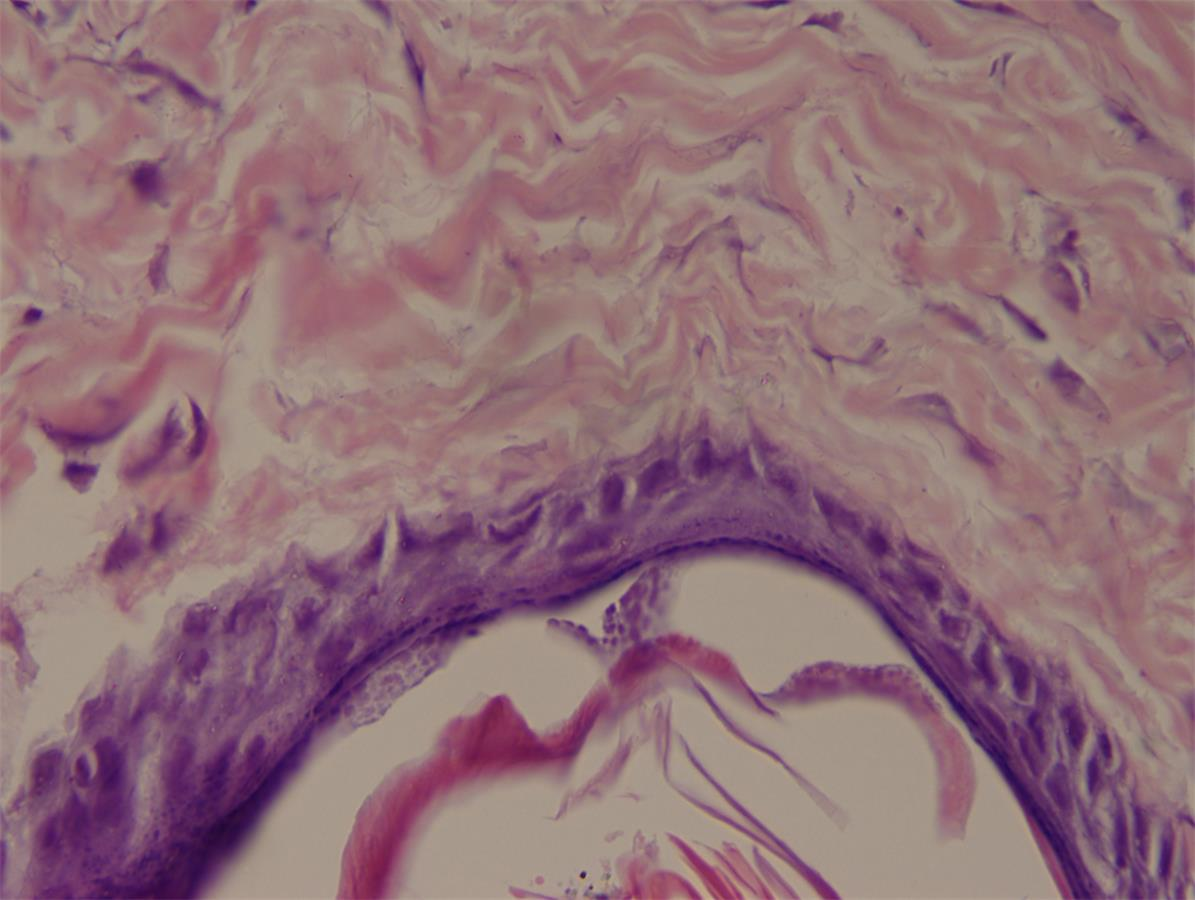

Supplement: Supplemental Information 2 — Representative images of granulation tissue sections. (A) Staining using Masson’s trichrome of ischemic and non-ischemic wounds in diabetic mice (×400). (B) HE staining of ischemic and non-ischemic wounds in diabetic mice (×400). (C) HE staining of ischemic and non-ischemic wounds in diabetic mice (×1,000). (D) Caspase-3 staining of ischemic and non-ischemic wounds in diabetic mice (×400). Scale bar = 100μm. (E) Duration of wound healing. [file peerj-09-11256-s002.zip › raw data 1/Figure 1C NONISCH-vehicle.png]

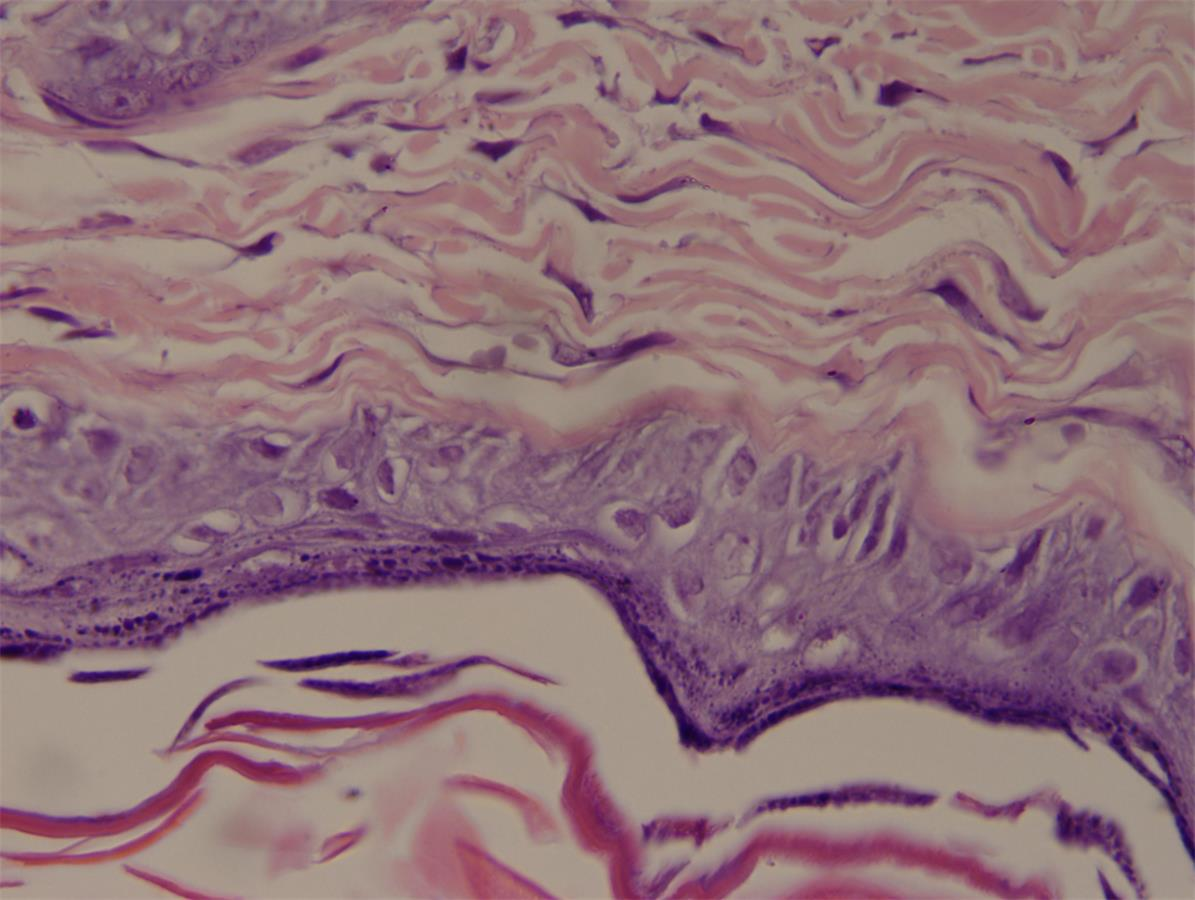

Supplement: Supplemental Information 2 — Representative images of granulation tissue sections. (A) Staining using Masson’s trichrome of ischemic and non-ischemic wounds in diabetic mice (×400). (B) HE staining of ischemic and non-ischemic wounds in diabetic mice (×400). (C) HE staining of ischemic and non-ischemic wounds in diabetic mice (×1,000). (D) Caspase-3 staining of ischemic and non-ischemic wounds in diabetic mice (×400). Scale bar = 100μm. (E) Duration of wound healing. [file peerj-09-11256-s002.zip › raw data 1/Figure 1C ISCH-fusion protein.png]

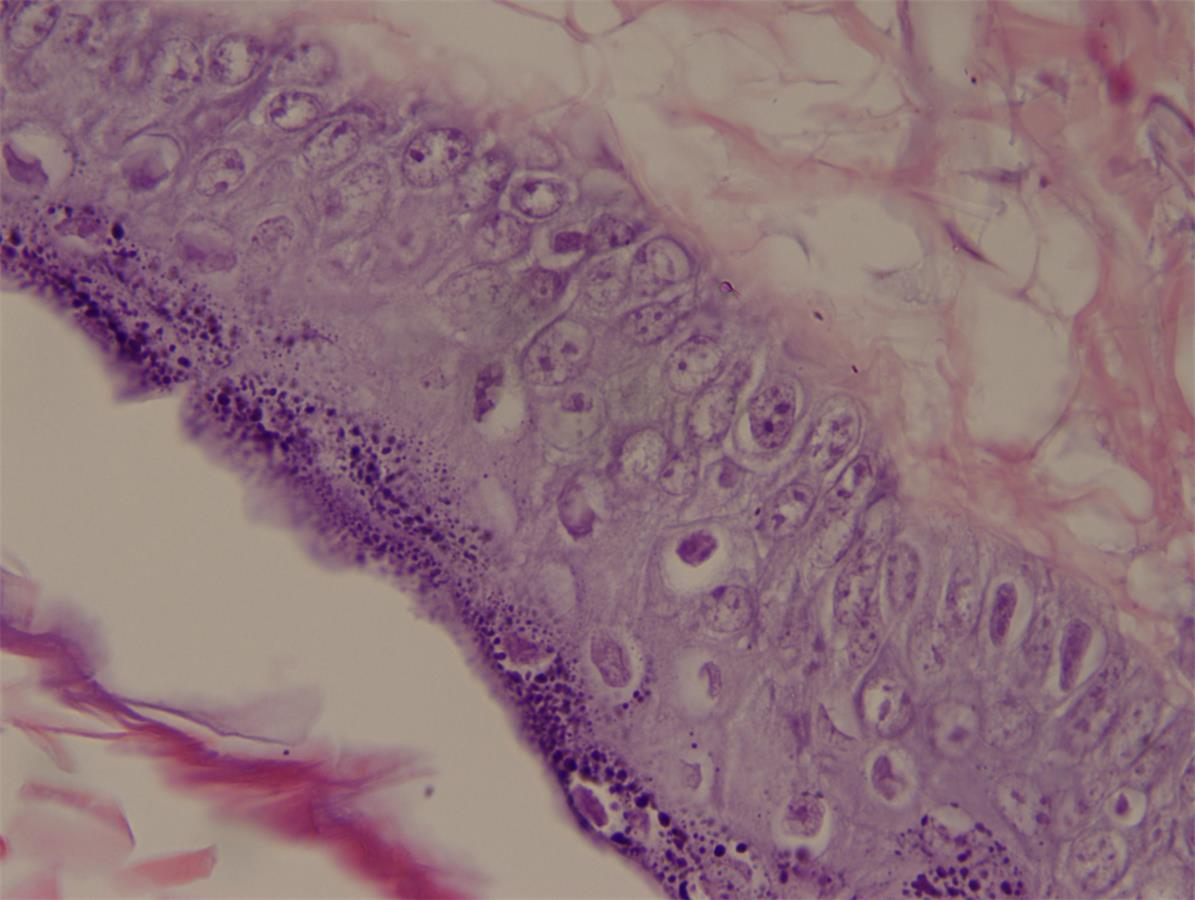

Supplement: Supplemental Information 2 — Representative images of granulation tissue sections. (A) Staining using Masson’s trichrome of ischemic and non-ischemic wounds in diabetic mice (×400). (B) HE staining of ischemic and non-ischemic wounds in diabetic mice (×400). (C) HE staining of ischemic and non-ischemic wounds in diabetic mice (×1,000). (D) Caspase-3 staining of ischemic and non-ischemic wounds in diabetic mice (×400). Scale bar = 100μm. (E) Duration of wound healing. [file peerj-09-11256-s002.zip › raw data 1/Figure 1C NONISCH-fusion protein.png]

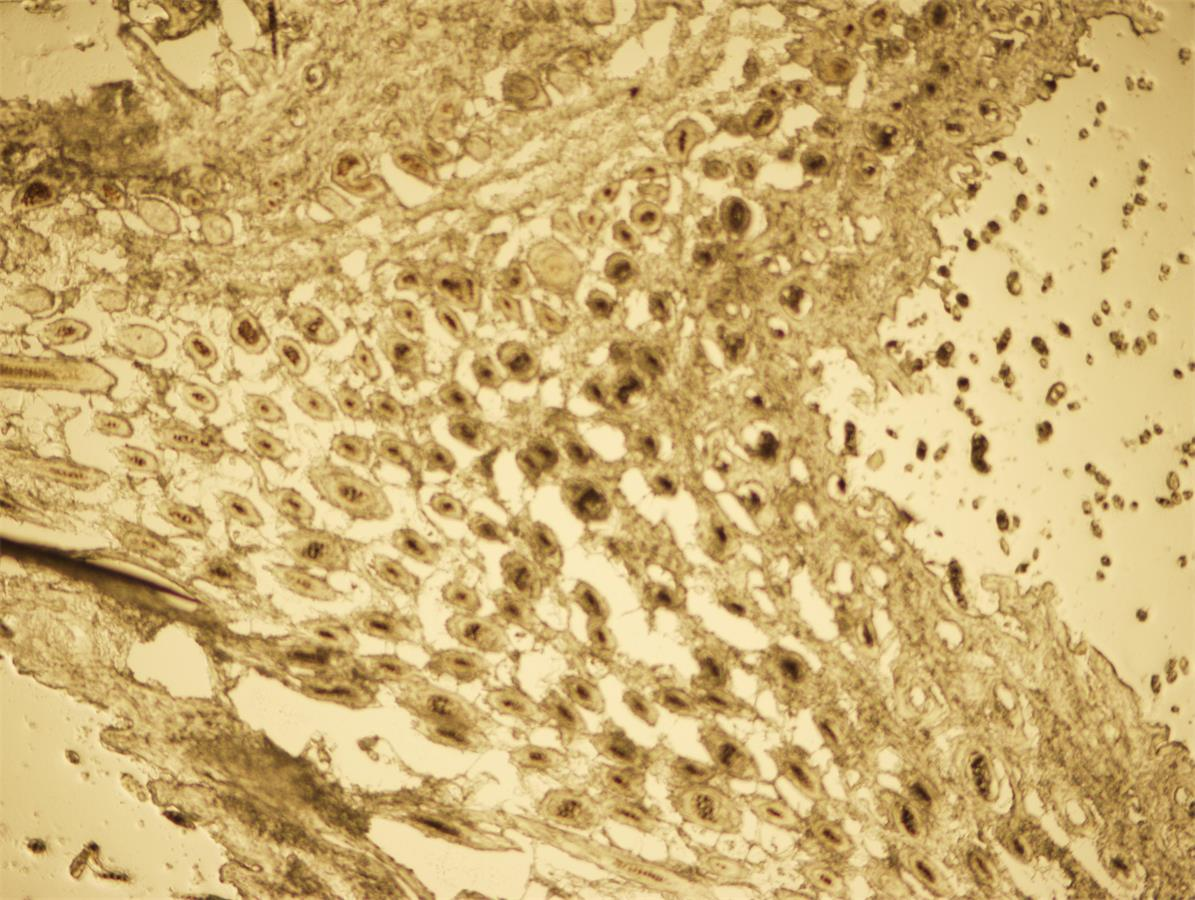

Supplement: Supplemental Information 2 — Representative images of granulation tissue sections. (A) Staining using Masson’s trichrome of ischemic and non-ischemic wounds in diabetic mice (×400). (B) HE staining of ischemic and non-ischemic wounds in diabetic mice (×400). (C) HE staining of ischemic and non-ischemic wounds in diabetic mice (×1,000). (D) Caspase-3 staining of ischemic and non-ischemic wounds in diabetic mice (×400). Scale bar = 100μm. (E) Duration of wound healing. [file peerj-09-11256-s002.zip › raw data 1/Figure 1D ISCH-vehicle.png]

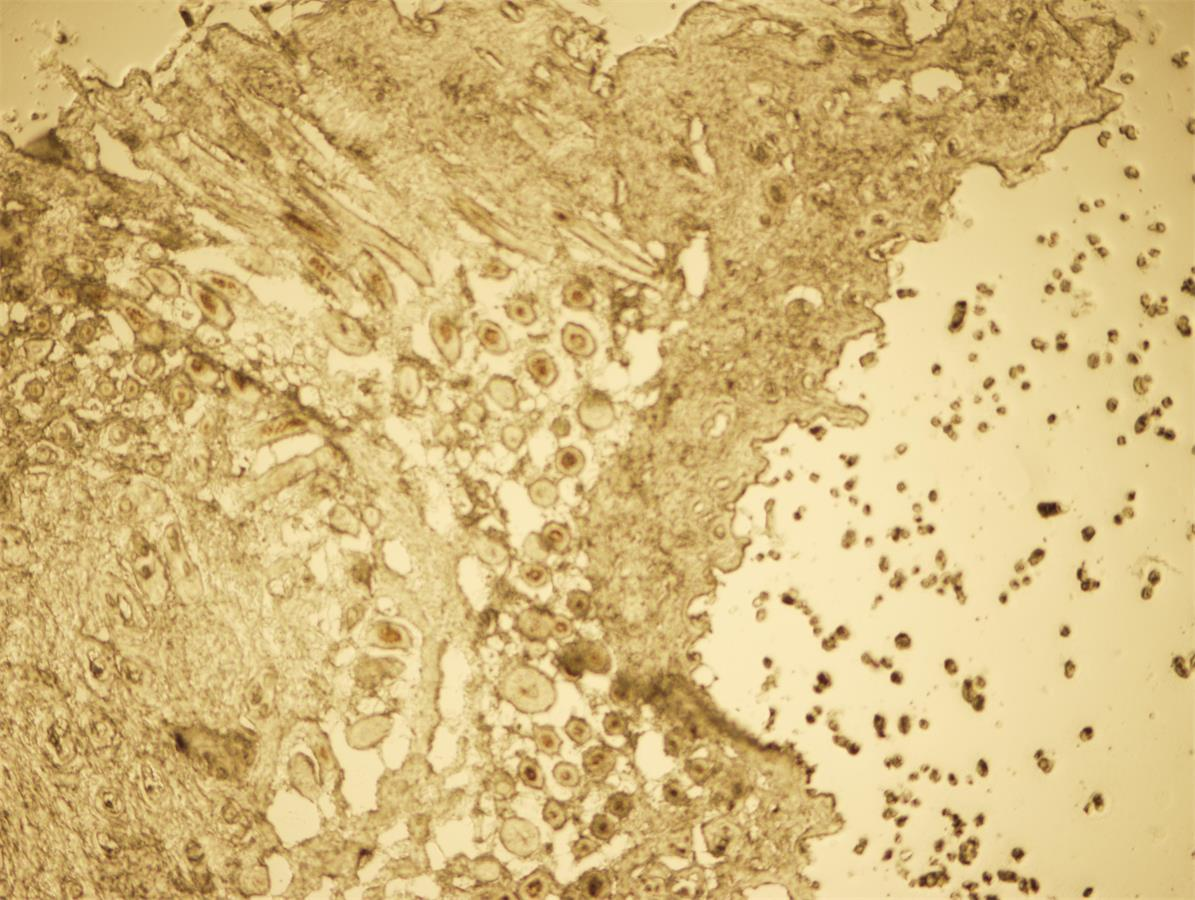

Supplement: Supplemental Information 2 — Representative images of granulation tissue sections. (A) Staining using Masson’s trichrome of ischemic and non-ischemic wounds in diabetic mice (×400). (B) HE staining of ischemic and non-ischemic wounds in diabetic mice (×400). (C) HE staining of ischemic and non-ischemic wounds in diabetic mice (×1,000). (D) Caspase-3 staining of ischemic and non-ischemic wounds in diabetic mice (×400). Scale bar = 100μm. (E) Duration of wound healing. [file peerj-09-11256-s002.zip › raw data 1/Figure 1D NONISCH-vehicle.png]

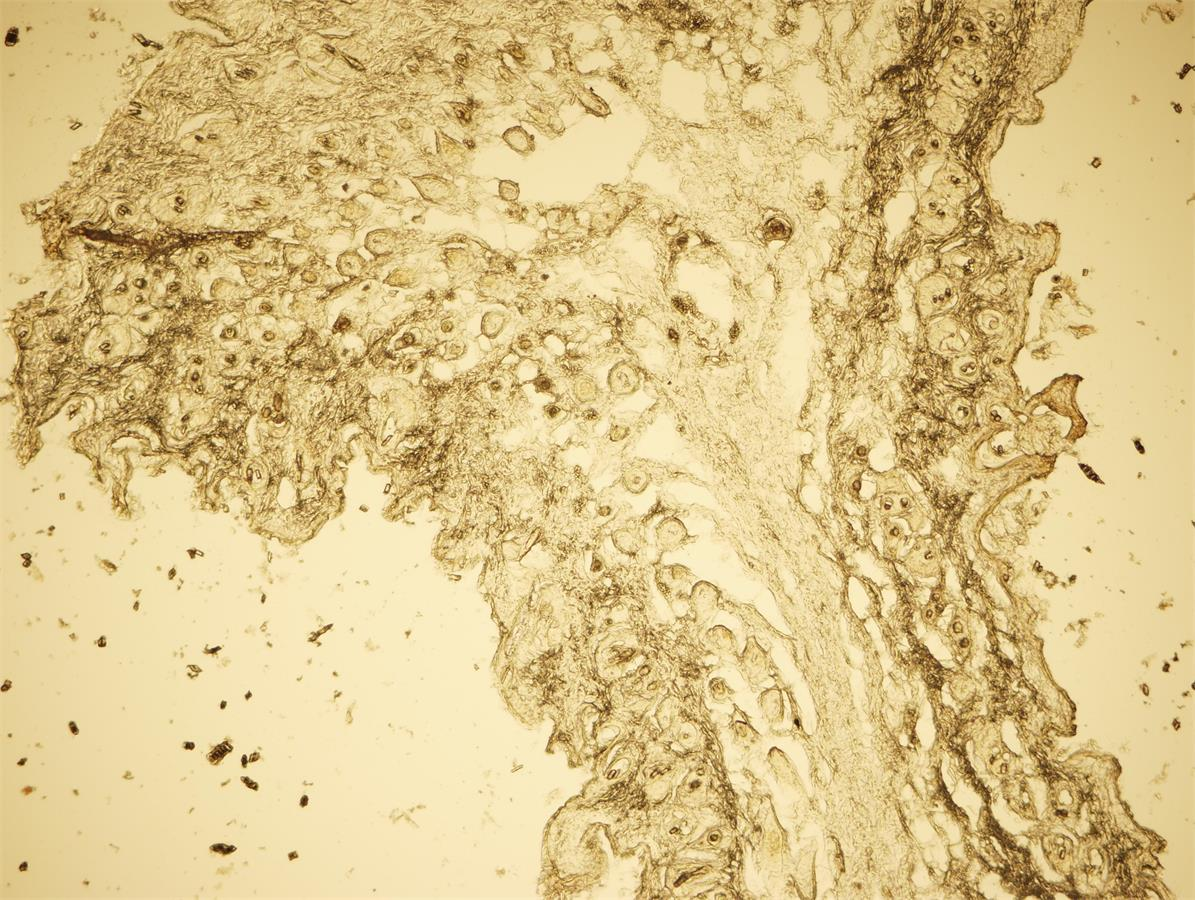

Supplement: Supplemental Information 2 — Representative images of granulation tissue sections. (A) Staining using Masson’s trichrome of ischemic and non-ischemic wounds in diabetic mice (×400). (B) HE staining of ischemic and non-ischemic wounds in diabetic mice (×400). (C) HE staining of ischemic and non-ischemic wounds in diabetic mice (×1,000). (D) Caspase-3 staining of ischemic and non-ischemic wounds in diabetic mice (×400). Scale bar = 100μm. (E) Duration of wound healing. [file peerj-09-11256-s002.zip › raw data 1/Figure 1D NONISCH-fusion protein.png]

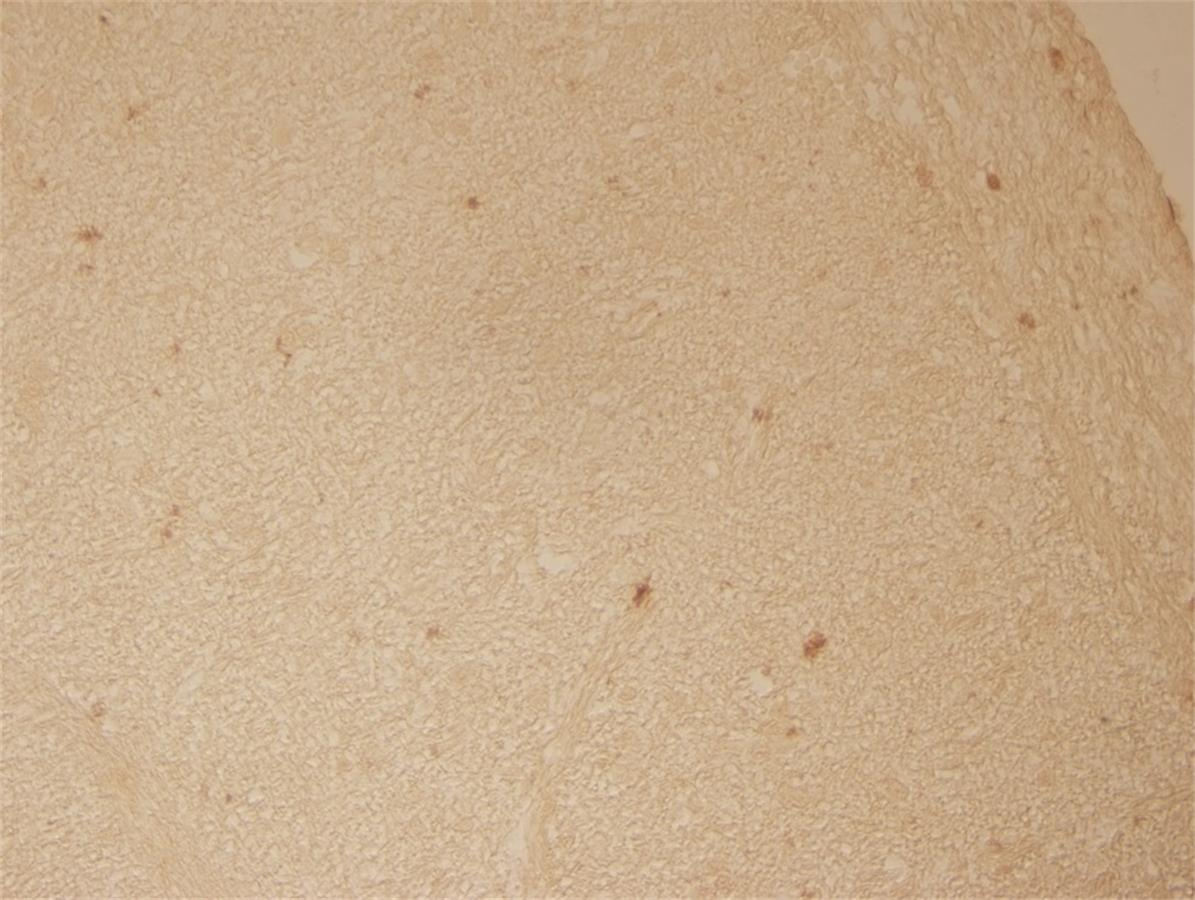

Supplement: Supplemental Information 2 — Representative images of granulation tissue sections. (A) Staining using Masson’s trichrome of ischemic and non-ischemic wounds in diabetic mice (×400). (B) HE staining of ischemic and non-ischemic wounds in diabetic mice (×400). (C) HE staining of ischemic and non-ischemic wounds in diabetic mice (×1,000). (D) Caspase-3 staining of ischemic and non-ischemic wounds in diabetic mice (×400). Scale bar = 100μm. (E) Duration of wound healing. [file peerj-09-11256-s002.zip › raw data 1/Figure 4D NONISCH-vehicle.png]

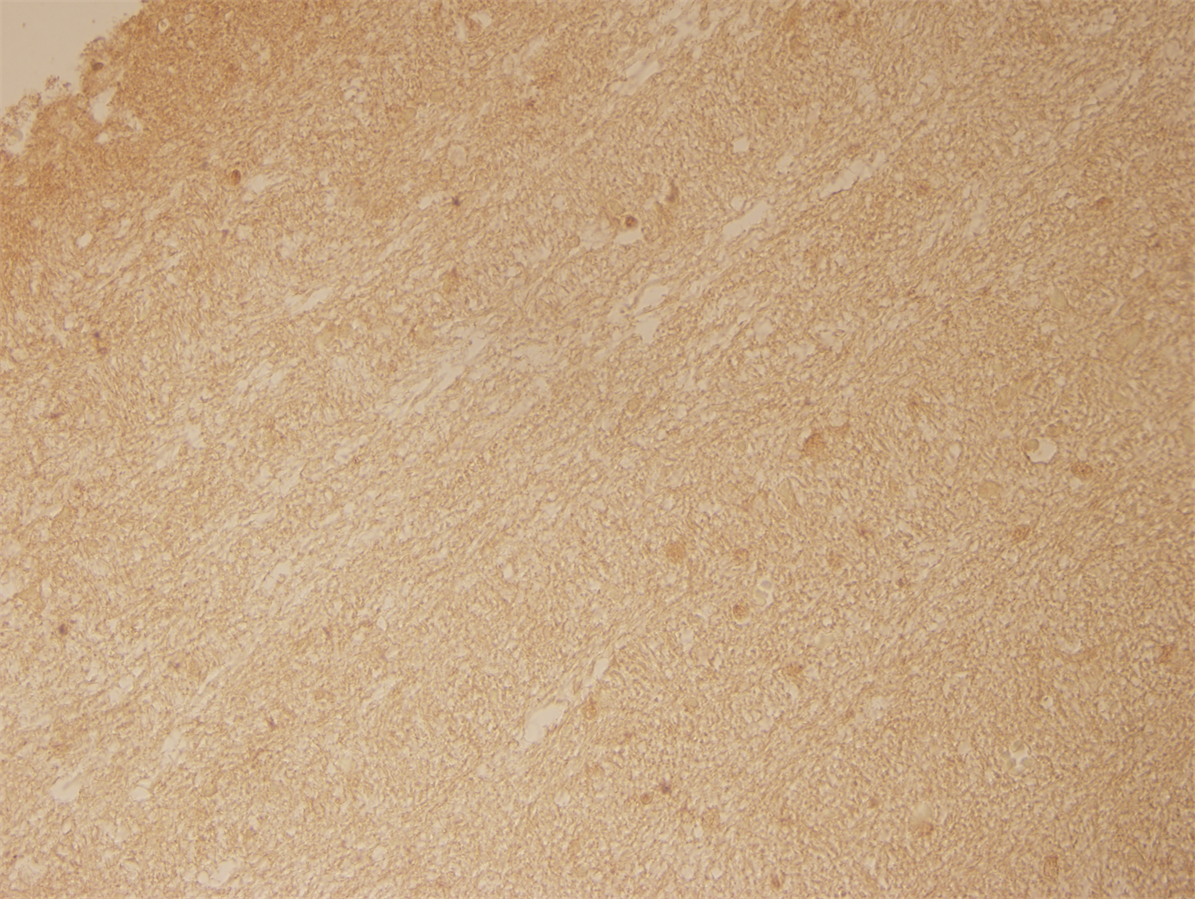

Supplement: Supplemental Information 2 — Representative images of granulation tissue sections. (A) Staining using Masson’s trichrome of ischemic and non-ischemic wounds in diabetic mice (×400). (B) HE staining of ischemic and non-ischemic wounds in diabetic mice (×400). (C) HE staining of ischemic and non-ischemic wounds in diabetic mice (×1,000). (D) Caspase-3 staining of ischemic and non-ischemic wounds in diabetic mice (×400). Scale bar = 100μm. (E) Duration of wound healing. [file peerj-09-11256-s002.zip › raw data 1/Figure 4D ISCH-fusion protein.png]

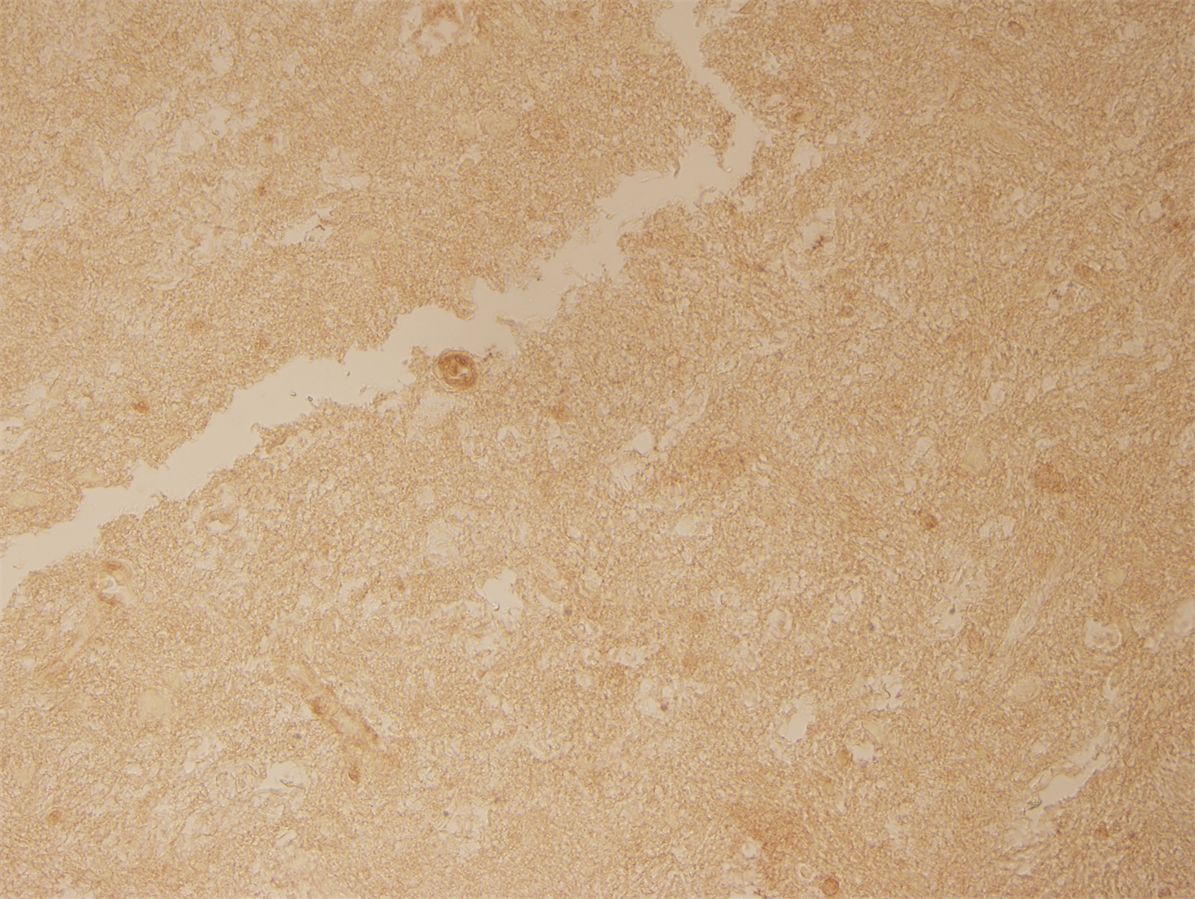

Supplement: Supplemental Information 2 — Representative images of granulation tissue sections. (A) Staining using Masson’s trichrome of ischemic and non-ischemic wounds in diabetic mice (×400). (B) HE staining of ischemic and non-ischemic wounds in diabetic mice (×400). (C) HE staining of ischemic and non-ischemic wounds in diabetic mice (×1,000). (D) Caspase-3 staining of ischemic and non-ischemic wounds in diabetic mice (×400). Scale bar = 100μm. (E) Duration of wound healing. [file peerj-09-11256-s002.zip › raw data 1/Figure 4D ISCH-vehicle.png]

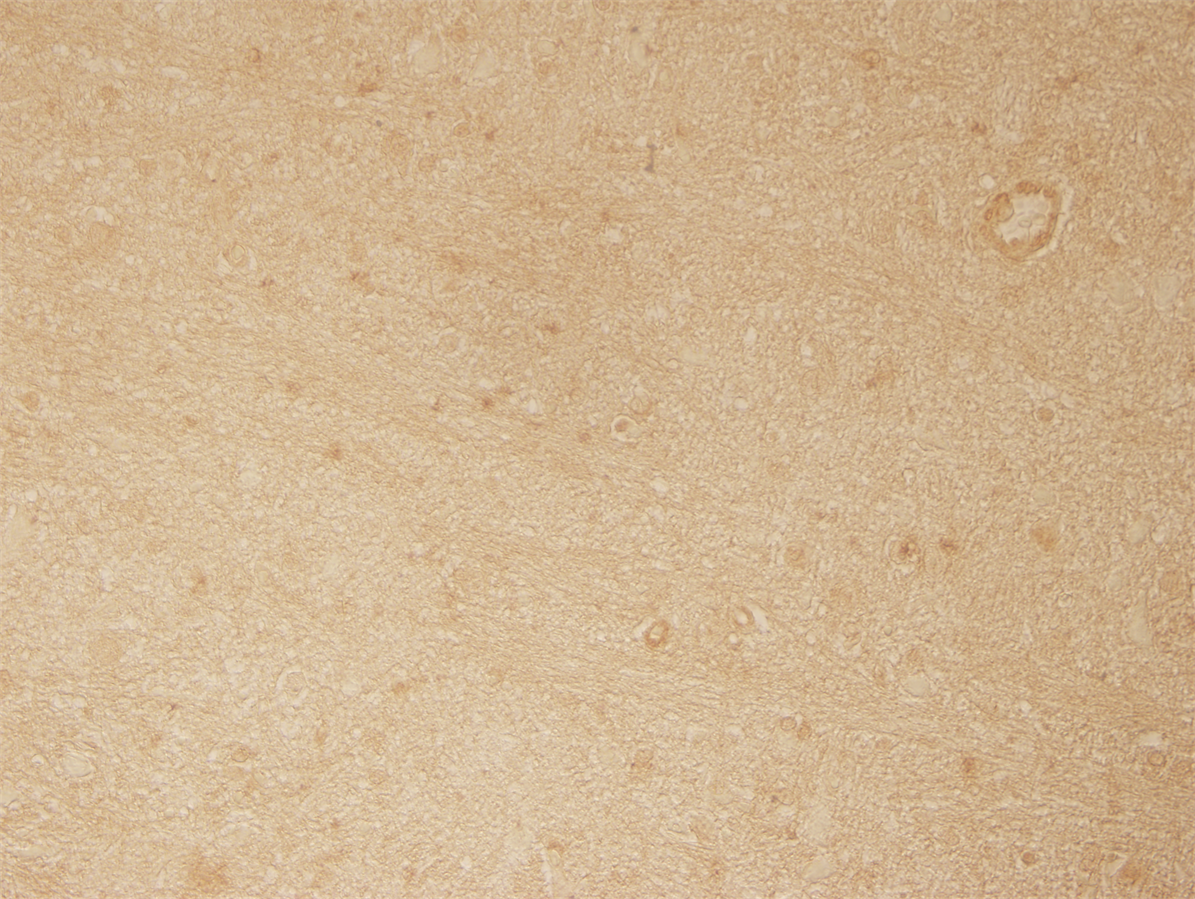

Supplement: Supplemental Information 2 — Representative images of granulation tissue sections. (A) Staining using Masson’s trichrome of ischemic and non-ischemic wounds in diabetic mice (×400). (B) HE staining of ischemic and non-ischemic wounds in diabetic mice (×400). (C) HE staining of ischemic and non-ischemic wounds in diabetic mice (×1,000). (D) Caspase-3 staining of ischemic and non-ischemic wounds in diabetic mice (×400). Scale bar = 100μm. (E) Duration of wound healing. [file peerj-09-11256-s002.zip › raw data 1/Figure 4D NONISCH-fusion protein.png]

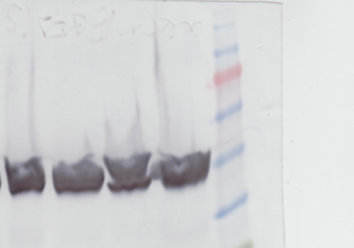

Supplement: Supplemental Information 2 — Representative images of granulation tissue sections. (A) Staining using Masson’s trichrome of ischemic and non-ischemic wounds in diabetic mice (×400). (B) HE staining of ischemic and non-ischemic wounds in diabetic mice (×400). (C) HE staining of ischemic and non-ischemic wounds in diabetic mice (×1,000). (D) Caspase-3 staining of ischemic and non-ischemic wounds in diabetic mice (×400). Scale bar = 100μm. (E) Duration of wound healing. [file peerj-09-11256-s002.zip › raw data 1/IKBɑ.png]

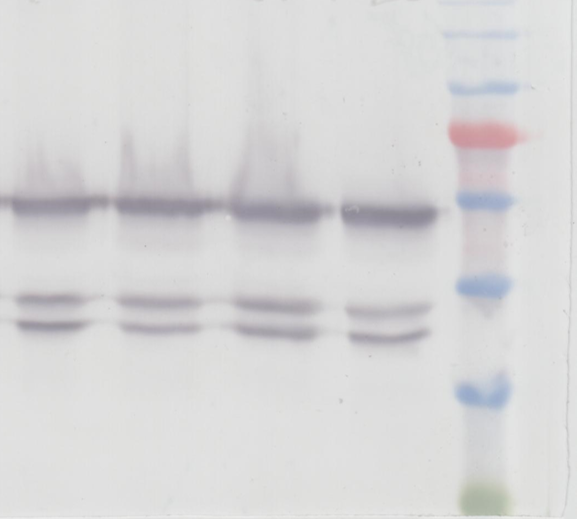

Supplement: Supplemental Information 2 — Representative images of granulation tissue sections. (A) Staining using Masson’s trichrome of ischemic and non-ischemic wounds in diabetic mice (×400). (B) HE staining of ischemic and non-ischemic wounds in diabetic mice (×400). (C) HE staining of ischemic and non-ischemic wounds in diabetic mice (×1,000). (D) Caspase-3 staining of ischemic and non-ischemic wounds in diabetic mice (×400). Scale bar = 100μm. (E) Duration of wound healing. [file peerj-09-11256-s002.zip › raw data 1/NF-KB (with ERK).png]

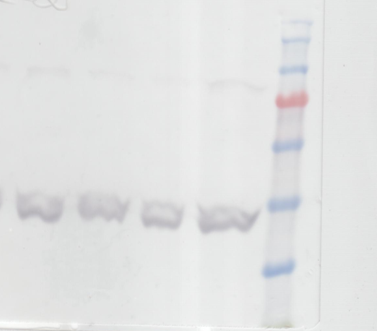

Supplement: Supplemental Information 2 — Representative images of granulation tissue sections. (A) Staining using Masson’s trichrome of ischemic and non-ischemic wounds in diabetic mice (×400). (B) HE staining of ischemic and non-ischemic wounds in diabetic mice (×400). (C) HE staining of ischemic and non-ischemic wounds in diabetic mice (×1,000). (D) Caspase-3 staining of ischemic and non-ischemic wounds in diabetic mice (×400). Scale bar = 100μm. (E) Duration of wound healing. [file peerj-09-11256-s002.zip › raw data 1/p-IKBɑ.png]

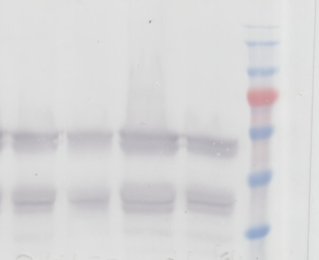

Supplement: Supplemental Information 2 — Representative images of granulation tissue sections. (A) Staining using Masson’s trichrome of ischemic and non-ischemic wounds in diabetic mice (×400). (B) HE staining of ischemic and non-ischemic wounds in diabetic mice (×400). (C) HE staining of ischemic and non-ischemic wounds in diabetic mice (×1,000). (D) Caspase-3 staining of ischemic and non-ischemic wounds in diabetic mice (×400). Scale bar = 100μm. (E) Duration of wound healing. [file peerj-09-11256-s002.zip › raw data 1/p-NF-KB (with p-ERK).png]

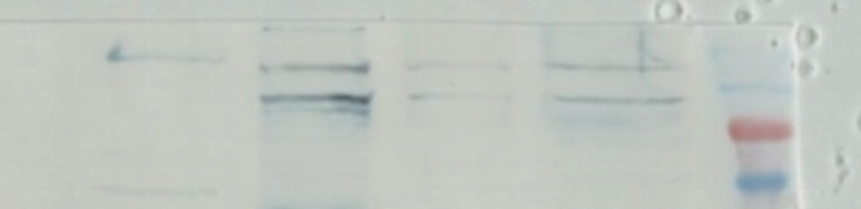

Supplement: Supplemental Information 2 — Representative images of granulation tissue sections. (A) Staining using Masson’s trichrome of ischemic and non-ischemic wounds in diabetic mice (×400). (B) HE staining of ischemic and non-ischemic wounds in diabetic mice (×400). (C) HE staining of ischemic and non-ischemic wounds in diabetic mice (×1,000). (D) Caspase-3 staining of ischemic and non-ischemic wounds in diabetic mice (×400). Scale bar = 100μm. (E) Duration of wound healing. [file peerj-09-11256-s002.zip › raw data 1/p-PDGFR.png]

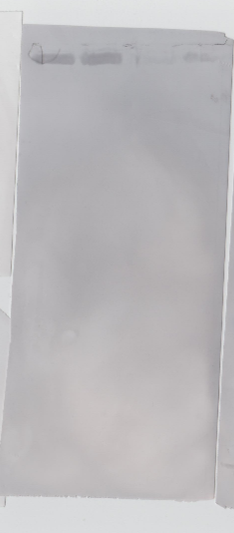

Supplement: Supplemental Information 2 — Representative images of granulation tissue sections. (A) Staining using Masson’s trichrome of ischemic and non-ischemic wounds in diabetic mice (×400). (B) HE staining of ischemic and non-ischemic wounds in diabetic mice (×400). (C) HE staining of ischemic and non-ischemic wounds in diabetic mice (×1,000). (D) Caspase-3 staining of ischemic and non-ischemic wounds in diabetic mice (×400). Scale bar = 100μm. (E) Duration of wound healing. [file peerj-09-11256-s002.zip › raw data 1/p-VEGFR.tif]

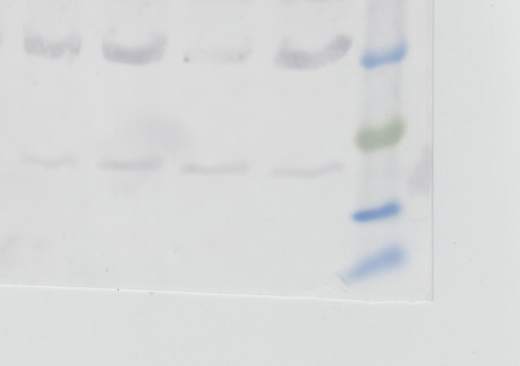

Supplement: Supplemental Information 2 — Representative images of granulation tissue sections. (A) Staining using Masson’s trichrome of ischemic and non-ischemic wounds in diabetic mice (×400). (B) HE staining of ischemic and non-ischemic wounds in diabetic mice (×400). (C) HE staining of ischemic and non-ischemic wounds in diabetic mice (×1,000). (D) Caspase-3 staining of ischemic and non-ischemic wounds in diabetic mice (×400). Scale bar = 100μm. (E) Duration of wound healing. [file peerj-09-11256-s002.zip › raw data 1/PDGF.png]

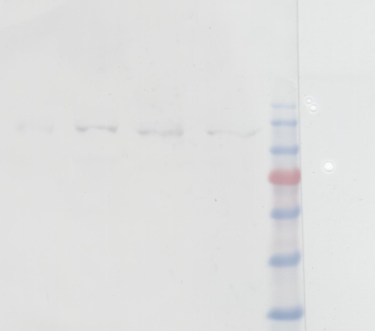

Supplement: Supplemental Information 2 — Representative images of granulation tissue sections. (A) Staining using Masson’s trichrome of ischemic and non-ischemic wounds in diabetic mice (×400). (B) HE staining of ischemic and non-ischemic wounds in diabetic mice (×400). (C) HE staining of ischemic and non-ischemic wounds in diabetic mice (×1,000). (D) Caspase-3 staining of ischemic and non-ischemic wounds in diabetic mice (×400). Scale bar = 100μm. (E) Duration of wound healing. [file peerj-09-11256-s002.zip › raw data 1/PDGFR.png]

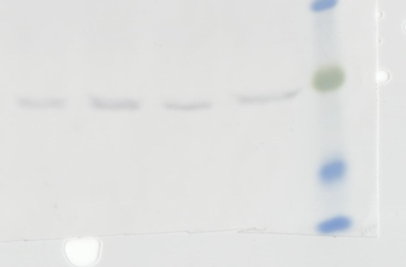

Supplement: Supplemental Information 2 — Representative images of granulation tissue sections. (A) Staining using Masson’s trichrome of ischemic and non-ischemic wounds in diabetic mice (×400). (B) HE staining of ischemic and non-ischemic wounds in diabetic mice (×400). (C) HE staining of ischemic and non-ischemic wounds in diabetic mice (×1,000). (D) Caspase-3 staining of ischemic and non-ischemic wounds in diabetic mice (×400). Scale bar = 100μm. (E) Duration of wound healing. [file peerj-09-11256-s002.zip › raw data 1/VEGF.png]

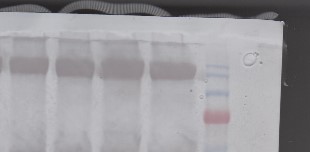

Supplement: Supplemental Information 2 — Representative images of granulation tissue sections. (A) Staining using Masson’s trichrome of ischemic and non-ischemic wounds in diabetic mice (×400). (B) HE staining of ischemic and non-ischemic wounds in diabetic mice (×400). (C) HE staining of ischemic and non-ischemic wounds in diabetic mice (×1,000). (D) Caspase-3 staining of ischemic and non-ischemic wounds in diabetic mice (×400). Scale bar = 100μm. (E) Duration of wound healing. [file peerj-09-11256-s002.zip › raw data 1/VERFR.jpg]

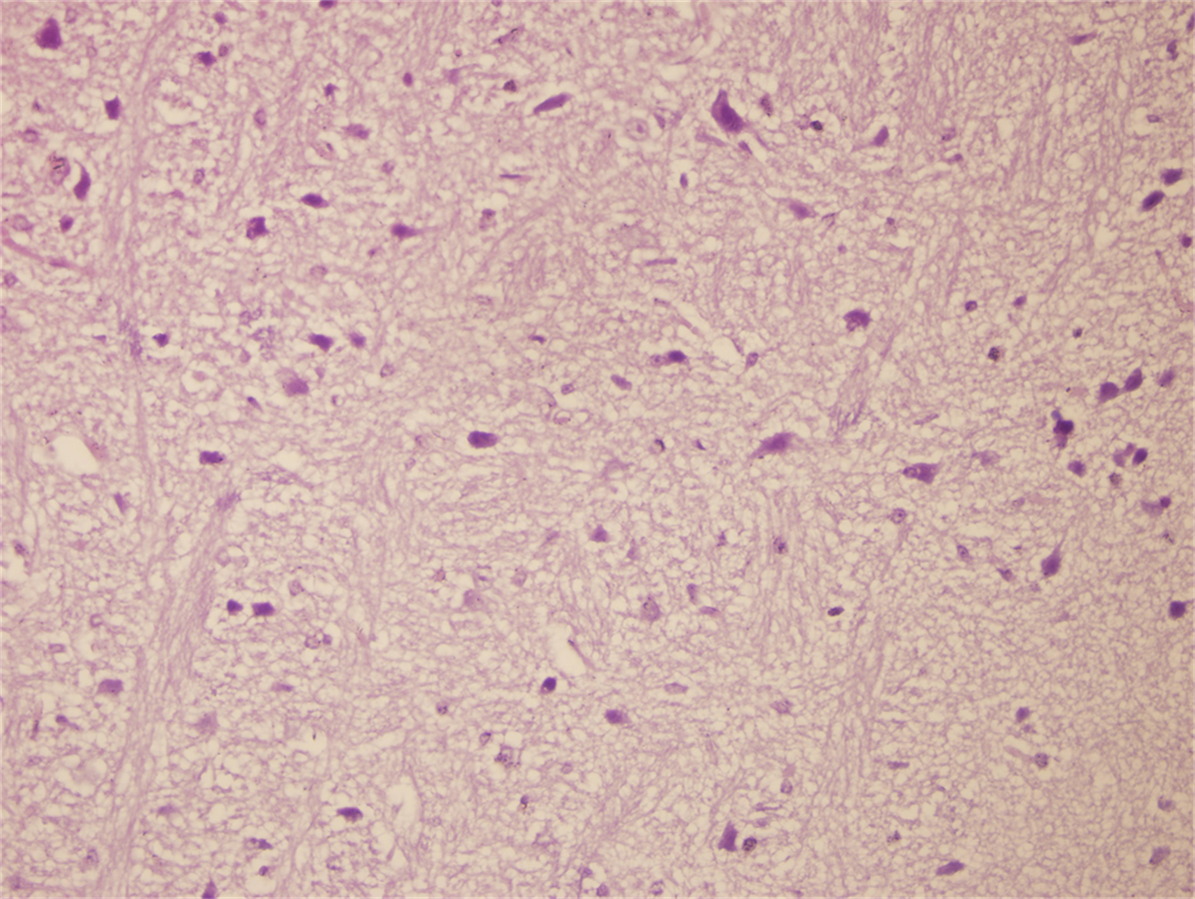

Supplement: Supplemental Information 3 — (A) Representative images of adductor muscle sections stained by HE (×400). (B) Representative images of adductor muscle sections stained by HE (×1,000). (C) Representative images of adductor muscle sections stained for CD34 (×400). (D) Representative images of adductor muscle sections stained for PDGF (×400). (E) Representative images of adductor muscle sections stained for Caspase 3 (×400). (F) Quantification of vascular density. Scale bar = 100μm. **P < 0.01 vs NONISCH; $P < 0.05 vs ISCH. [file peerj-09-11256-s003.zip › raw data 2/Figure 4A ISCH-fusion protein.png]

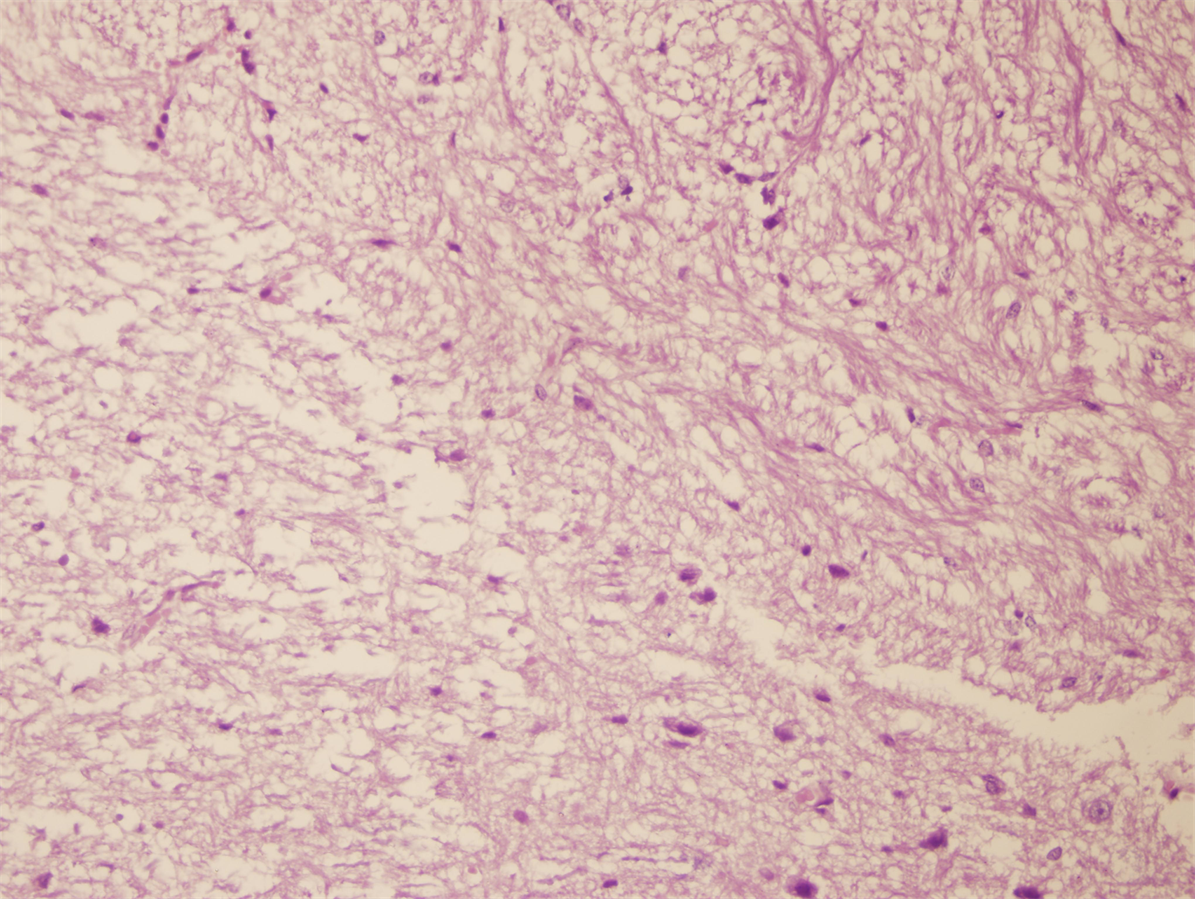

Supplement: Supplemental Information 3 — (A) Representative images of adductor muscle sections stained by HE (×400). (B) Representative images of adductor muscle sections stained by HE (×1,000). (C) Representative images of adductor muscle sections stained for CD34 (×400). (D) Representative images of adductor muscle sections stained for PDGF (×400). (E) Representative images of adductor muscle sections stained for Caspase 3 (×400). (F) Quantification of vascular density. Scale bar = 100μm. **P < 0.01 vs NONISCH; $P < 0.05 vs ISCH. [file peerj-09-11256-s003.zip › raw data 2/Figure 4A ISCH-vehicle.png]

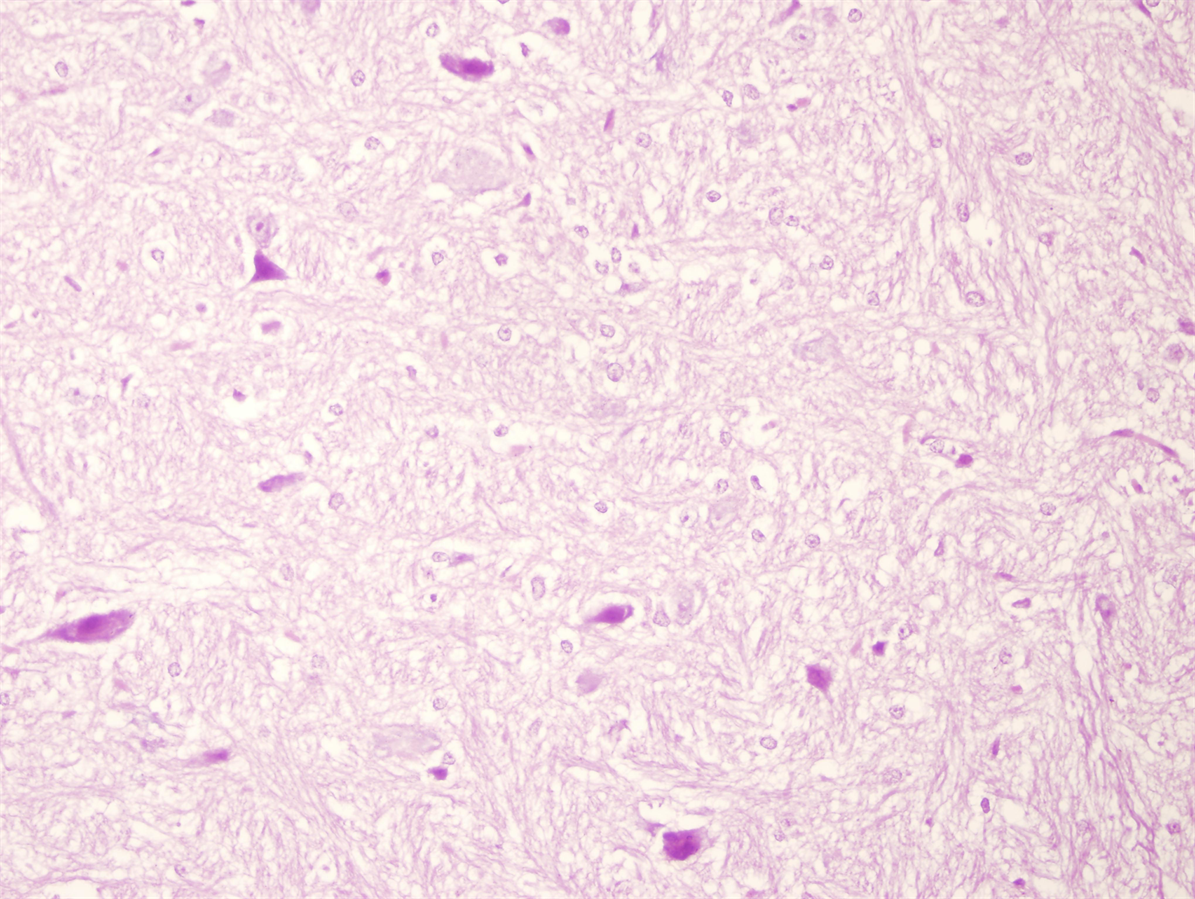

Supplement: Supplemental Information 3 — (A) Representative images of adductor muscle sections stained by HE (×400). (B) Representative images of adductor muscle sections stained by HE (×1,000). (C) Representative images of adductor muscle sections stained for CD34 (×400). (D) Representative images of adductor muscle sections stained for PDGF (×400). (E) Representative images of adductor muscle sections stained for Caspase 3 (×400). (F) Quantification of vascular density. Scale bar = 100μm. **P < 0.01 vs NONISCH; $P < 0.05 vs ISCH. [file peerj-09-11256-s003.zip › raw data 2/Figure 4A NONISCH-fusion protein.png]

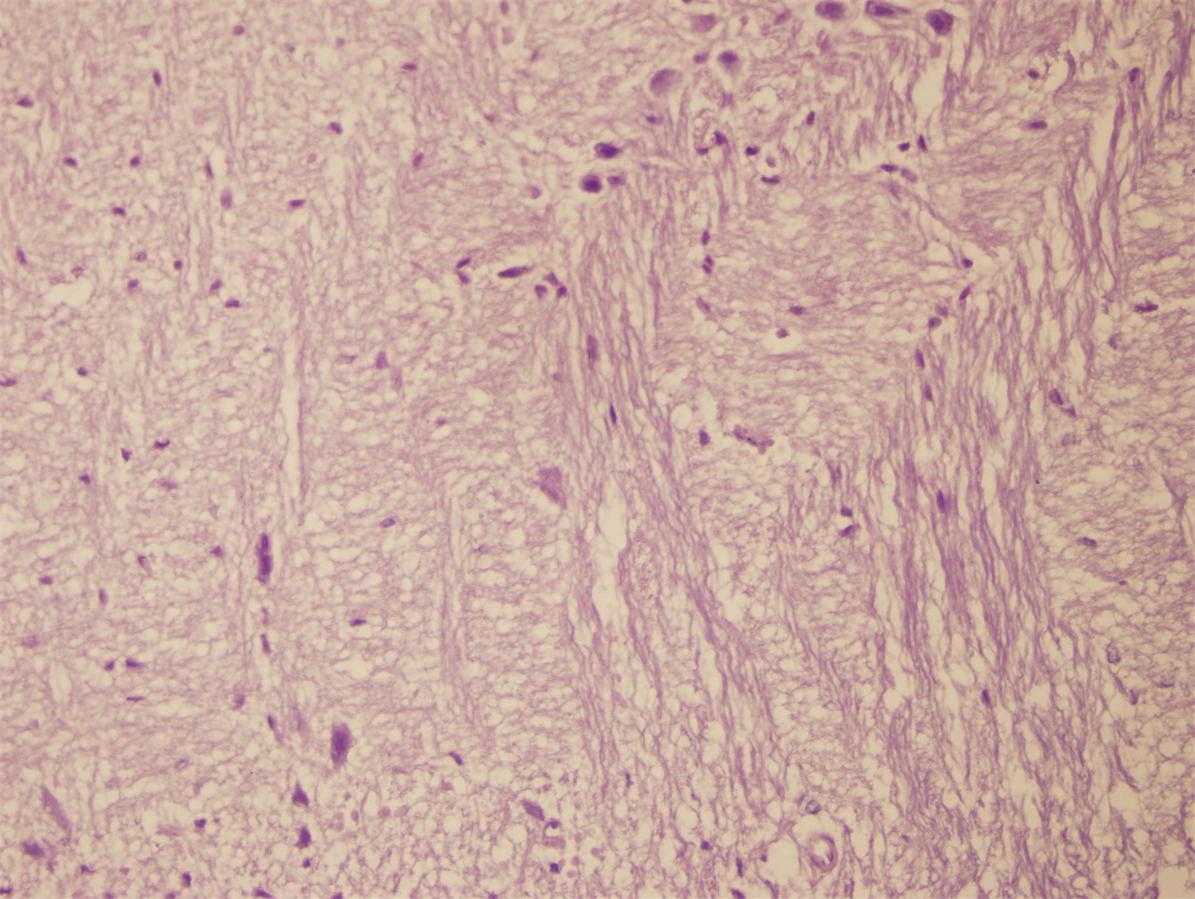

Supplement: Supplemental Information 3 — (A) Representative images of adductor muscle sections stained by HE (×400). (B) Representative images of adductor muscle sections stained by HE (×1,000). (C) Representative images of adductor muscle sections stained for CD34 (×400). (D) Representative images of adductor muscle sections stained for PDGF (×400). (E) Representative images of adductor muscle sections stained for Caspase 3 (×400). (F) Quantification of vascular density. Scale bar = 100μm. **P < 0.01 vs NONISCH; $P < 0.05 vs ISCH. [file peerj-09-11256-s003.zip › raw data 2/Figure 4A NONISCH-vehicle.png]

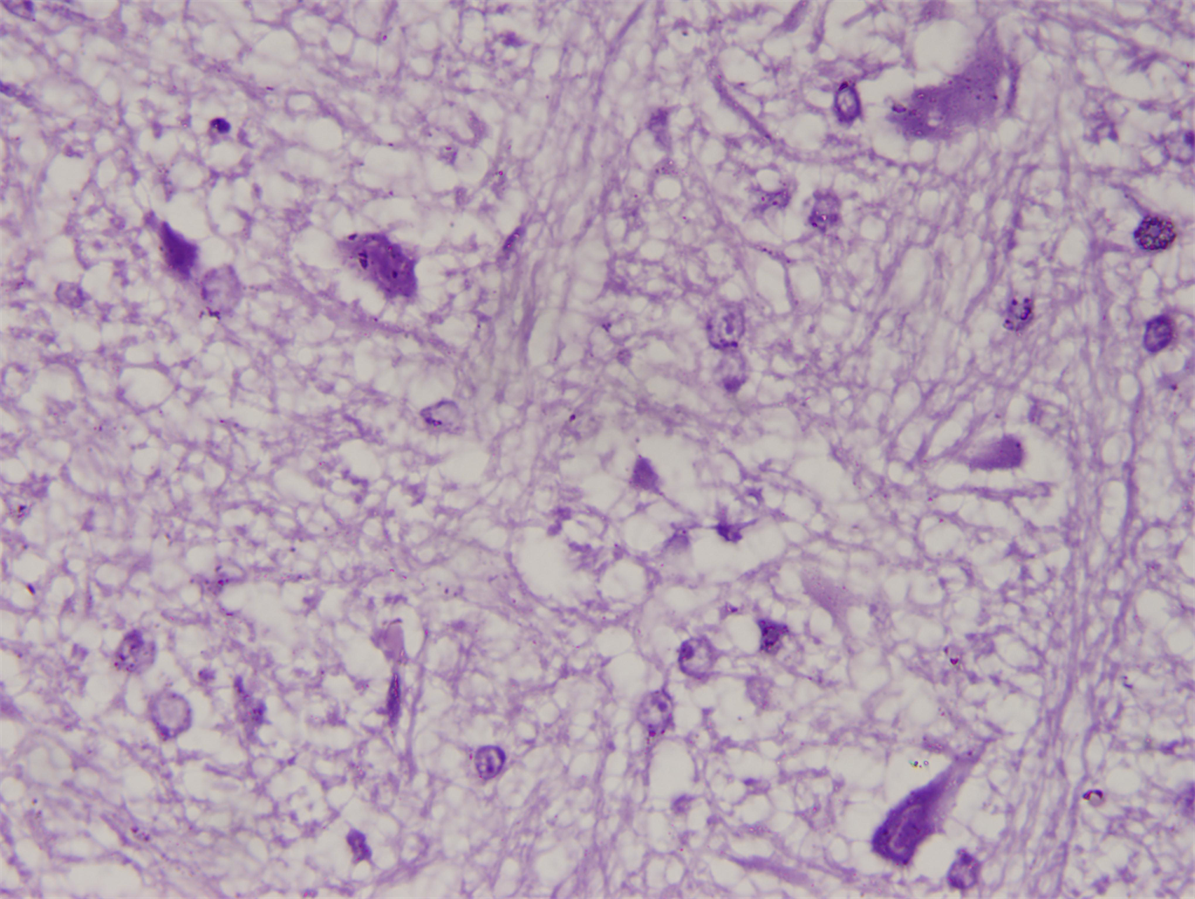

Supplement: Supplemental Information 3 — (A) Representative images of adductor muscle sections stained by HE (×400). (B) Representative images of adductor muscle sections stained by HE (×1,000). (C) Representative images of adductor muscle sections stained for CD34 (×400). (D) Representative images of adductor muscle sections stained for PDGF (×400). (E) Representative images of adductor muscle sections stained for Caspase 3 (×400). (F) Quantification of vascular density. Scale bar = 100μm. **P < 0.01 vs NONISCH; $P < 0.05 vs ISCH. [file peerj-09-11256-s003.zip › raw data 2/Figure 4B ISCH-fusion protein.png]

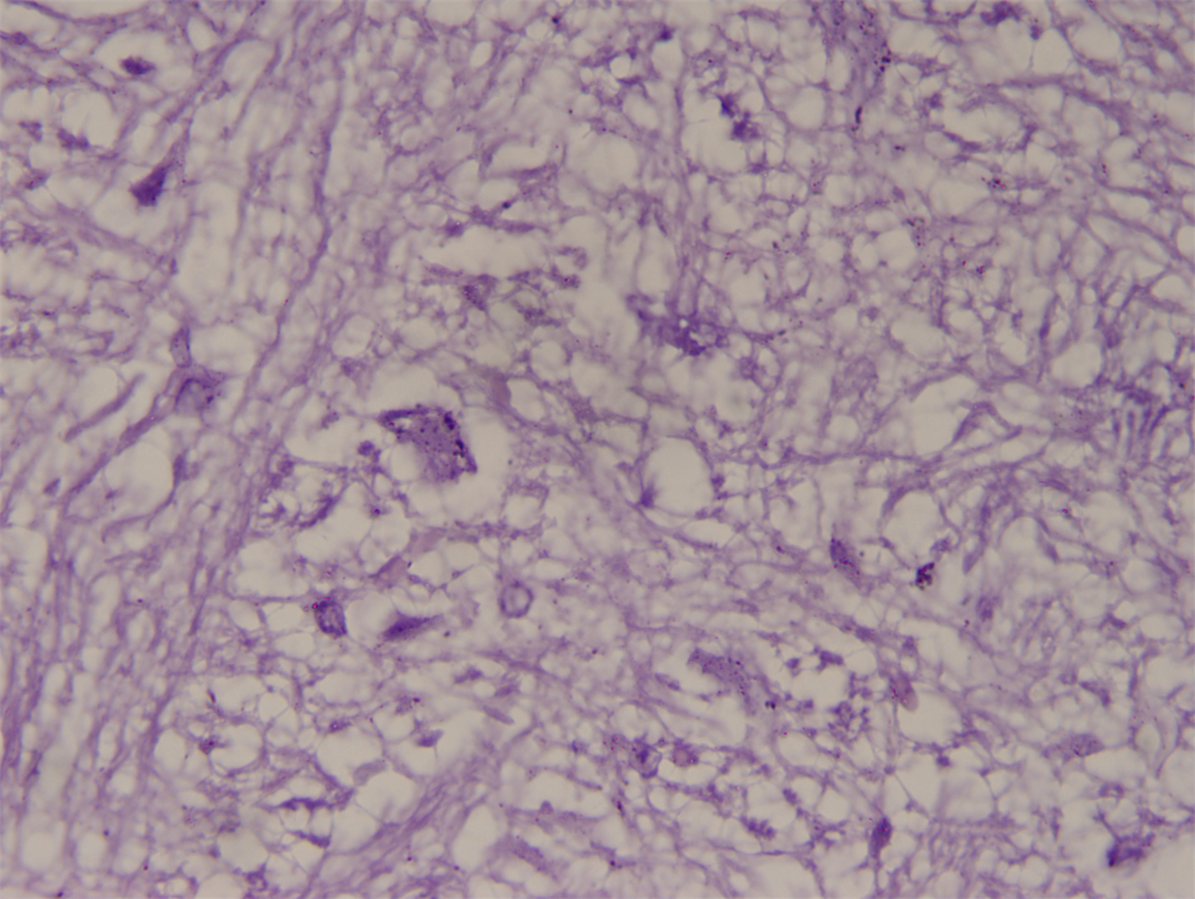

Supplement: Supplemental Information 3 — (A) Representative images of adductor muscle sections stained by HE (×400). (B) Representative images of adductor muscle sections stained by HE (×1,000). (C) Representative images of adductor muscle sections stained for CD34 (×400). (D) Representative images of adductor muscle sections stained for PDGF (×400). (E) Representative images of adductor muscle sections stained for Caspase 3 (×400). (F) Quantification of vascular density. Scale bar = 100μm. **P < 0.01 vs NONISCH; $P < 0.05 vs ISCH. [file peerj-09-11256-s003.zip › raw data 2/Figure 4B ISCH-vehicle.png]

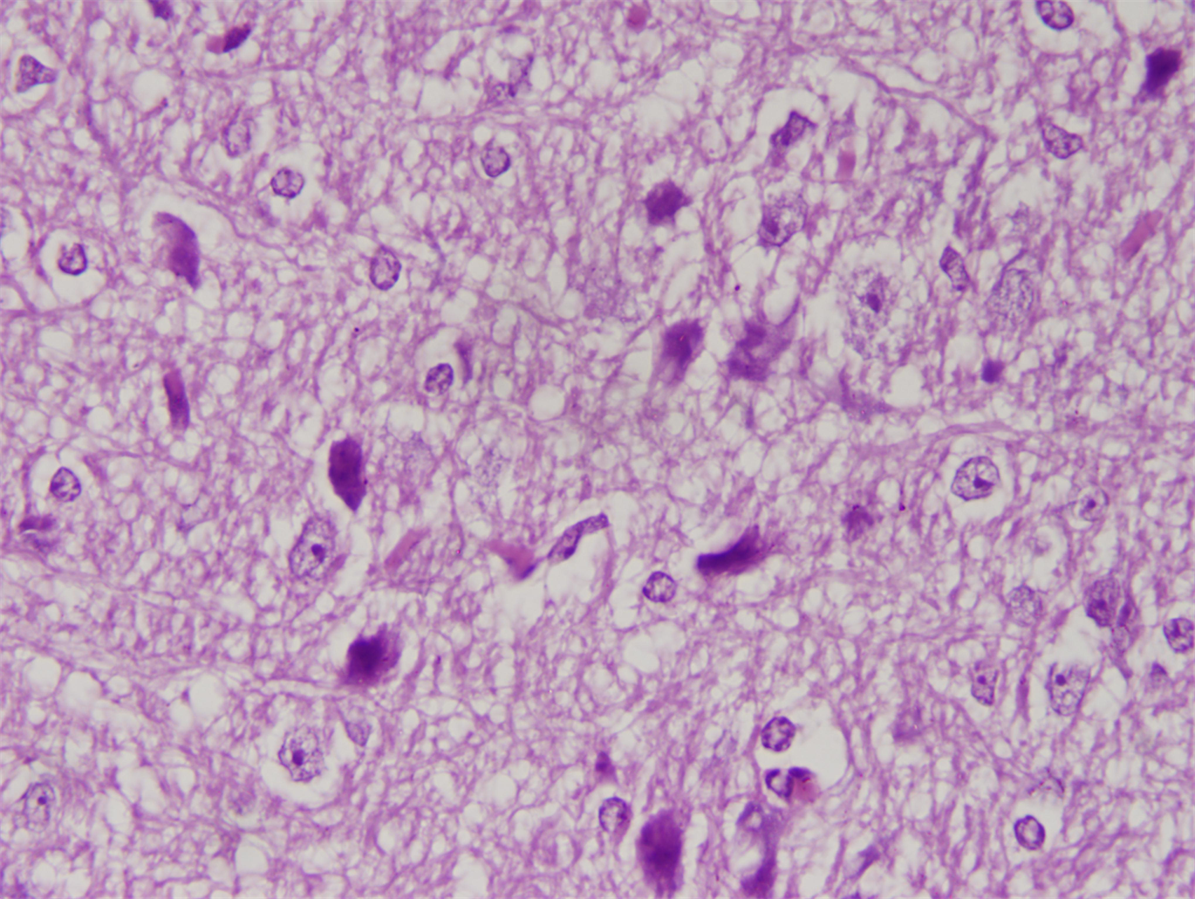

Supplement: Supplemental Information 3 — (A) Representative images of adductor muscle sections stained by HE (×400). (B) Representative images of adductor muscle sections stained by HE (×1,000). (C) Representative images of adductor muscle sections stained for CD34 (×400). (D) Representative images of adductor muscle sections stained for PDGF (×400). (E) Representative images of adductor muscle sections stained for Caspase 3 (×400). (F) Quantification of vascular density. Scale bar = 100μm. **P < 0.01 vs NONISCH; $P < 0.05 vs ISCH. [file peerj-09-11256-s003.zip › raw data 2/Figure 4B NONISCH-fusion protein.png]

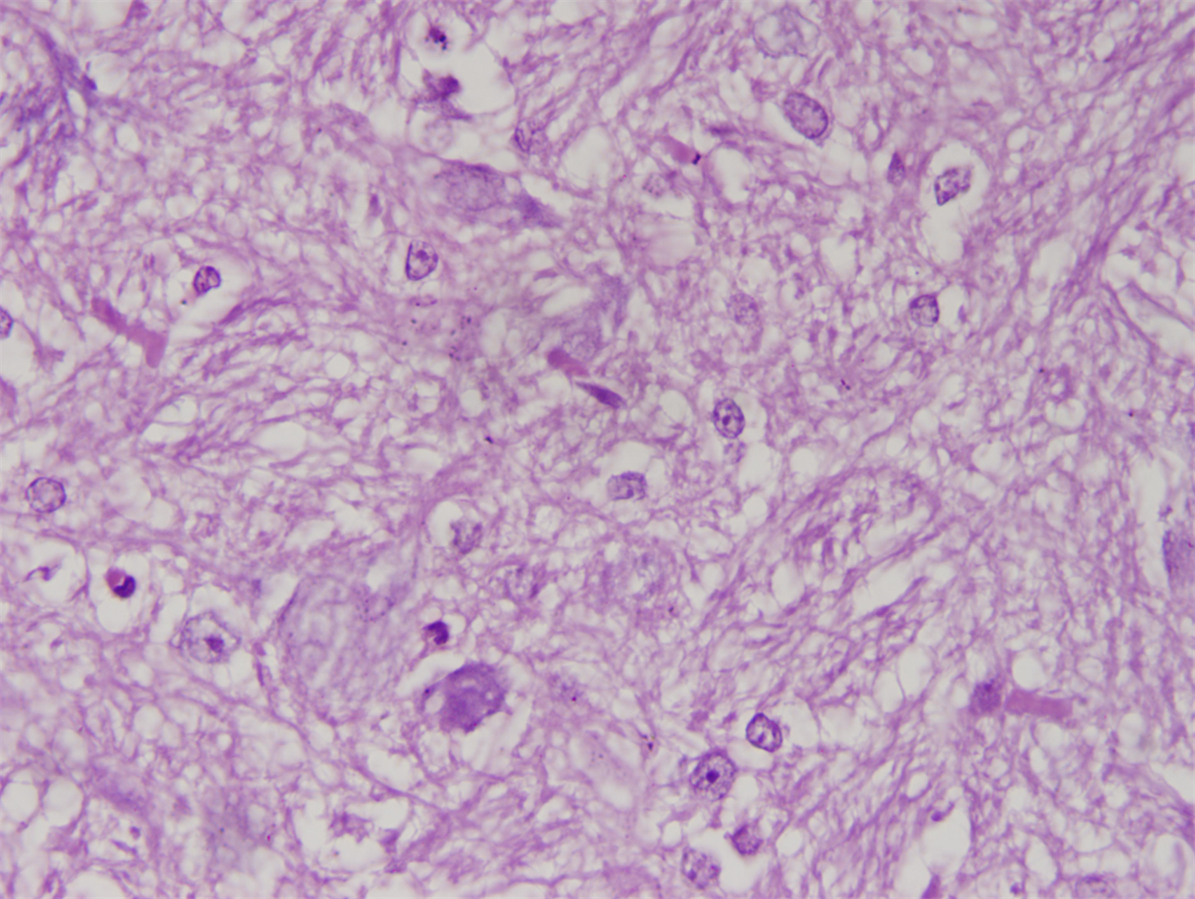

Supplement: Supplemental Information 3 — (A) Representative images of adductor muscle sections stained by HE (×400). (B) Representative images of adductor muscle sections stained by HE (×1,000). (C) Representative images of adductor muscle sections stained for CD34 (×400). (D) Representative images of adductor muscle sections stained for PDGF (×400). (E) Representative images of adductor muscle sections stained for Caspase 3 (×400). (F) Quantification of vascular density. Scale bar = 100μm. **P < 0.01 vs NONISCH; $P < 0.05 vs ISCH. [file peerj-09-11256-s003.zip › raw data 2/Figure 4B NONISCH-vehicle.png]

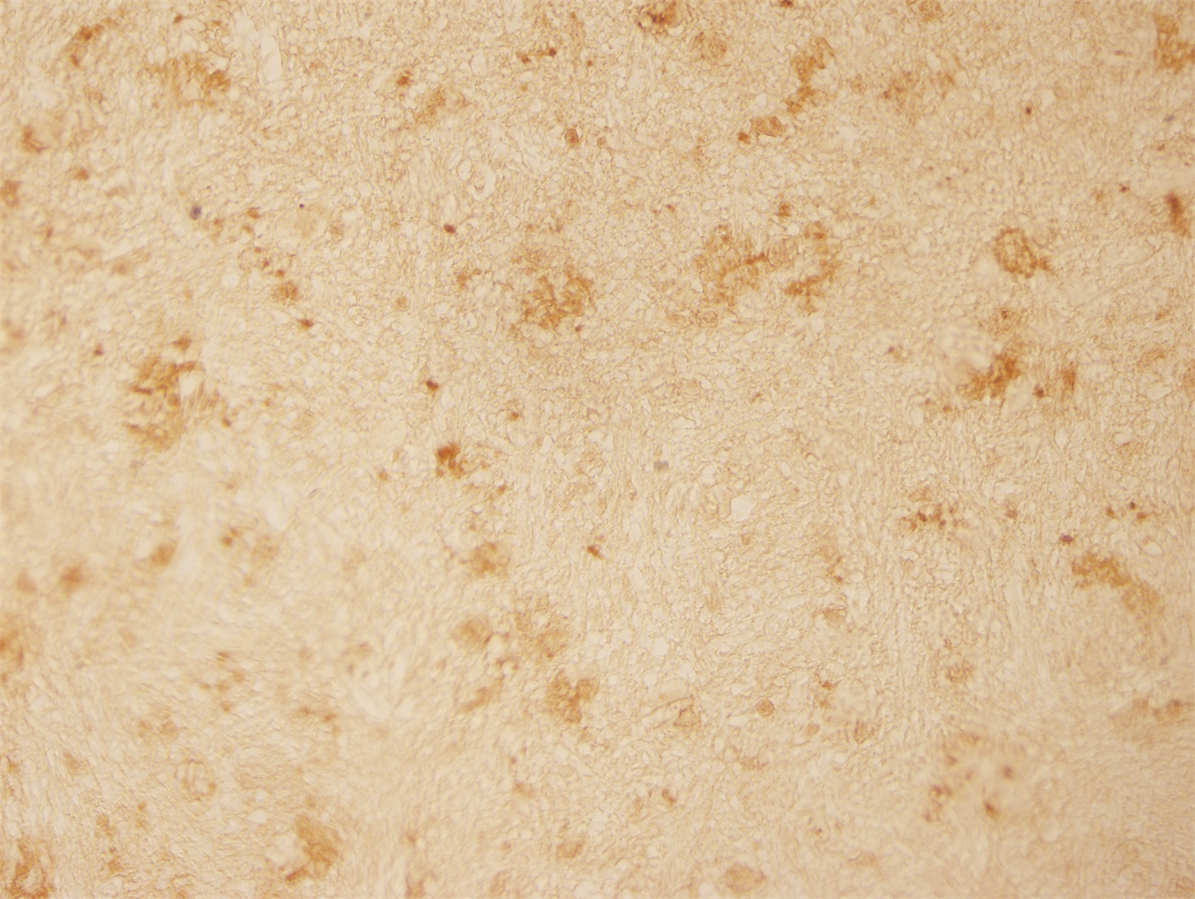

Supplement: Supplemental Information 3 — (A) Representative images of adductor muscle sections stained by HE (×400). (B) Representative images of adductor muscle sections stained by HE (×1,000). (C) Representative images of adductor muscle sections stained for CD34 (×400). (D) Representative images of adductor muscle sections stained for PDGF (×400). (E) Representative images of adductor muscle sections stained for Caspase 3 (×400). (F) Quantification of vascular density. Scale bar = 100μm. **P < 0.01 vs NONISCH; $P < 0.05 vs ISCH. [file peerj-09-11256-s003.zip › raw data 2/Figure 4C ISCH-fusion protein.png]

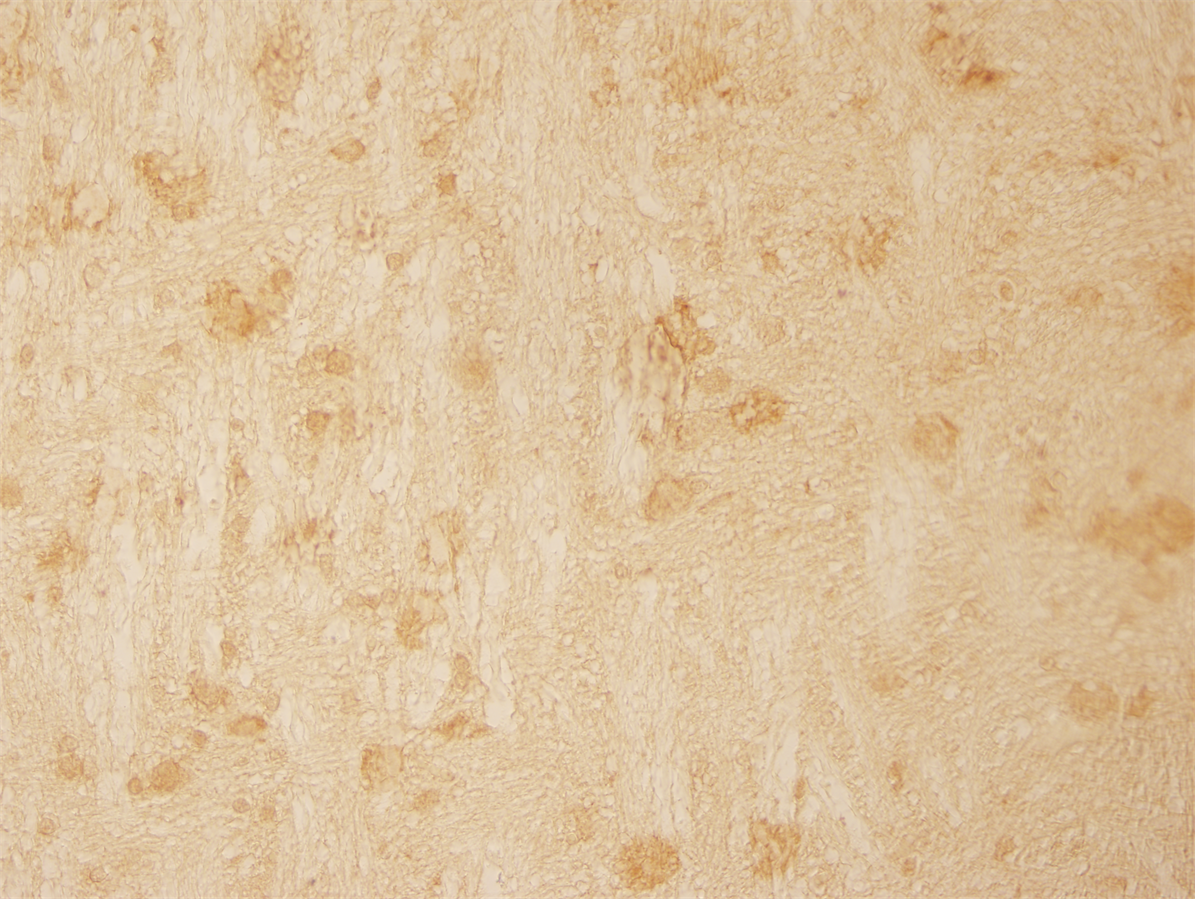

Supplement: Supplemental Information 3 — (A) Representative images of adductor muscle sections stained by HE (×400). (B) Representative images of adductor muscle sections stained by HE (×1,000). (C) Representative images of adductor muscle sections stained for CD34 (×400). (D) Representative images of adductor muscle sections stained for PDGF (×400). (E) Representative images of adductor muscle sections stained for Caspase 3 (×400). (F) Quantification of vascular density. Scale bar = 100μm. **P < 0.01 vs NONISCH; $P < 0.05 vs ISCH. [file peerj-09-11256-s003.zip › raw data 2/Figure 4C ISCH-vehicle.png]

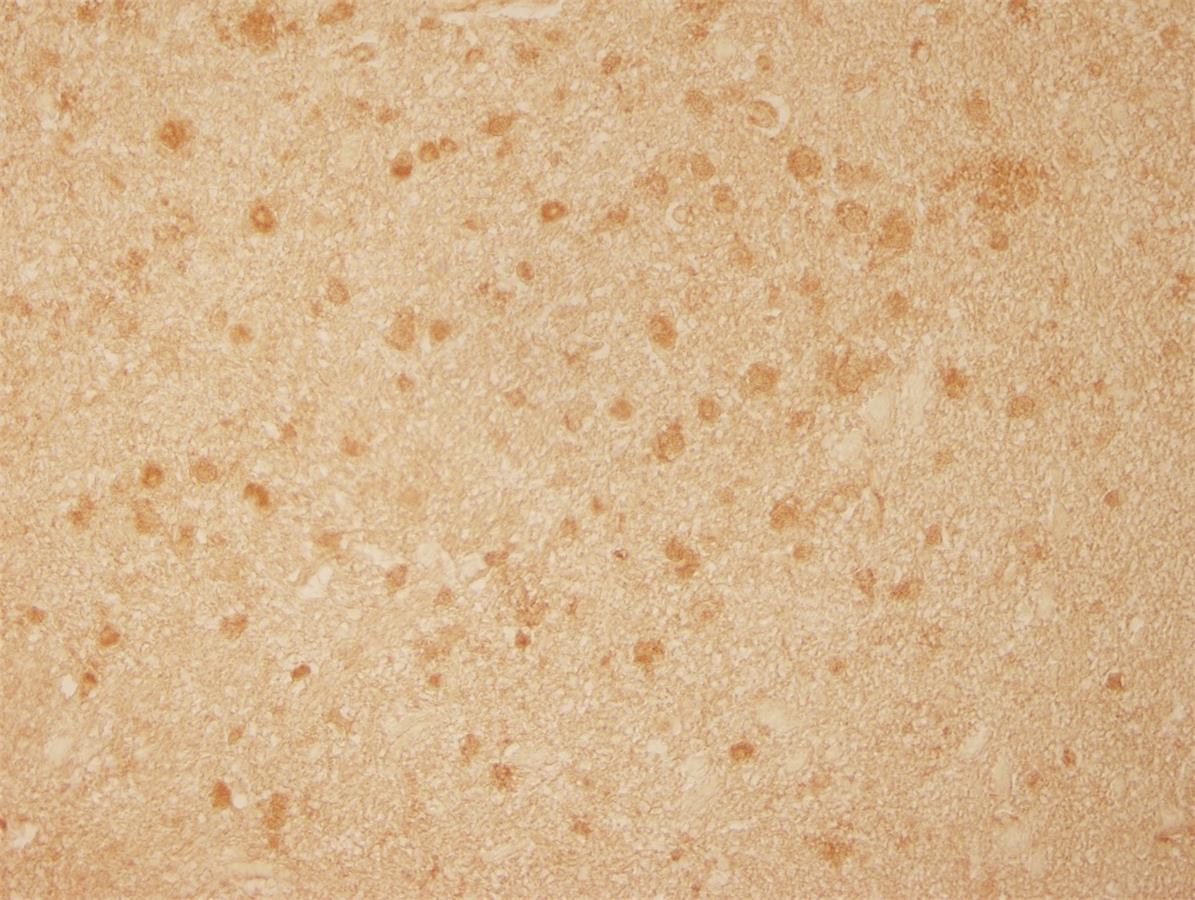

Supplement: Supplemental Information 3 — (A) Representative images of adductor muscle sections stained by HE (×400). (B) Representative images of adductor muscle sections stained by HE (×1,000). (C) Representative images of adductor muscle sections stained for CD34 (×400). (D) Representative images of adductor muscle sections stained for PDGF (×400). (E) Representative images of adductor muscle sections stained for Caspase 3 (×400). (F) Quantification of vascular density. Scale bar = 100μm. **P < 0.01 vs NONISCH; $P < 0.05 vs ISCH. [file peerj-09-11256-s003.zip › raw data 2/Figure 4C NONISCH-fusion protein.png]

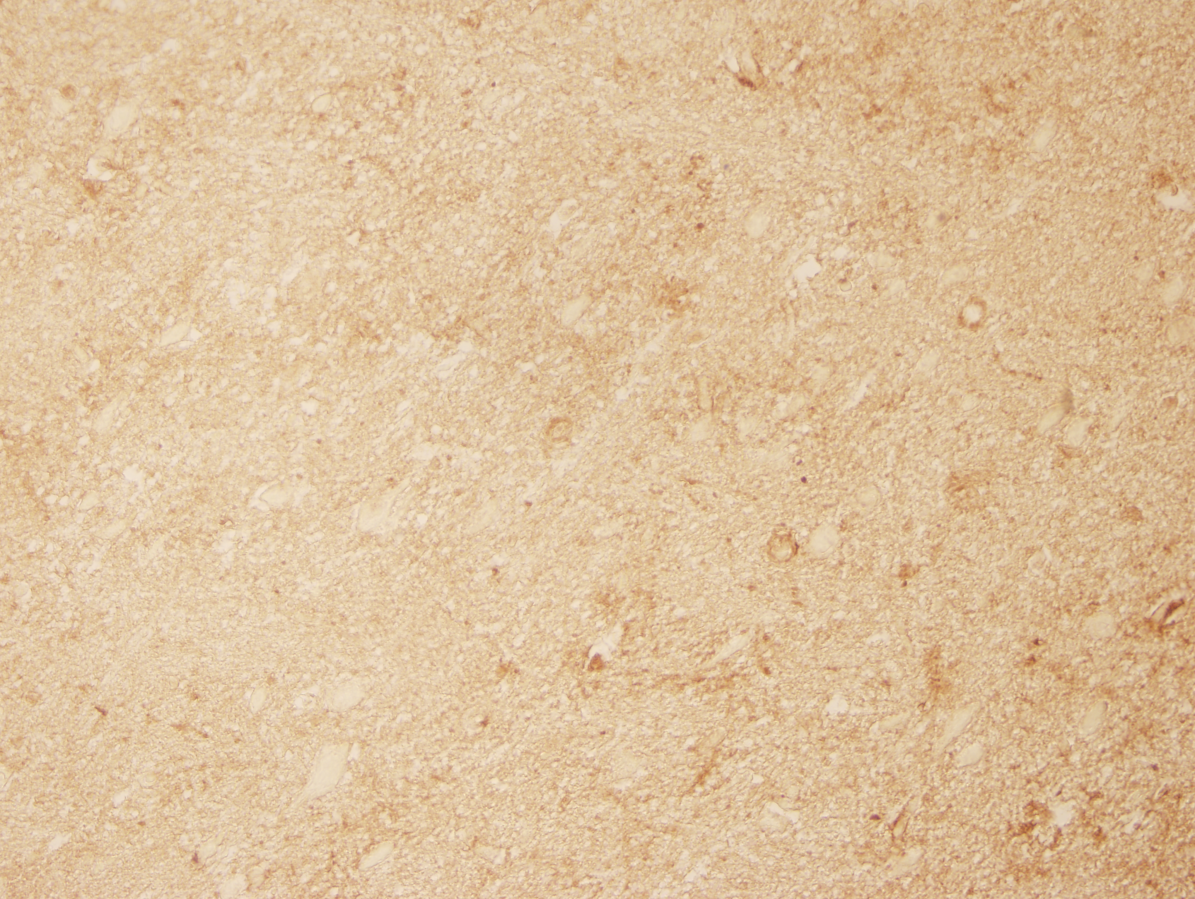

Supplement: Supplemental Information 3 — (A) Representative images of adductor muscle sections stained by HE (×400). (B) Representative images of adductor muscle sections stained by HE (×1,000). (C) Representative images of adductor muscle sections stained for CD34 (×400). (D) Representative images of adductor muscle sections stained for PDGF (×400). (E) Representative images of adductor muscle sections stained for Caspase 3 (×400). (F) Quantification of vascular density. Scale bar = 100μm. **P < 0.01 vs NONISCH; $P < 0.05 vs ISCH. [file peerj-09-11256-s003.zip › raw data 2/Figure 4C NONISCH-vehicle.png]

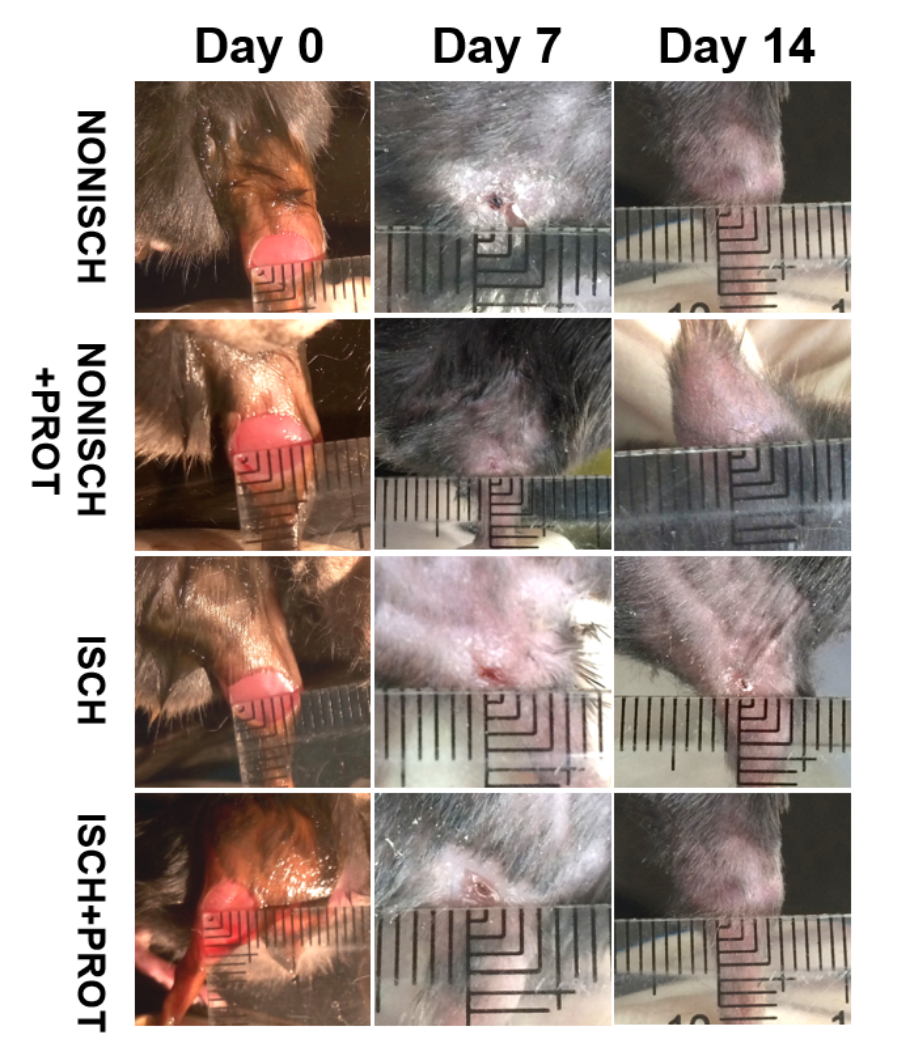

Supplement: Supplemental Information 4 [file peerj-09-11256-s004.png]
